# Comparison of macrocyclic and acyclic chelators for gallium-68 radiolabelling†
†Electronic supplementary information (ESI) available. CCDC 1564603. For ESI and crystallographic data in CIF or other electronic format see DOI: 10.1039/c7ra09076e


**DOI:** 10.1039/c7ra09076e

**Published:** 2017-10-25

**Authors:** Maria Iris Tsionou, Caroline E. Knapp, Calum A. Foley, Catherine R. Munteanu, Andrew Cakebread, Cinzia Imberti, Thomas R. Eykyn, Jennifer D. Young, Brett M. Paterson, Philip J. Blower, Michelle T. Ma

**Affiliations:** a King's College London, Division of Imaging Sciences and Biomedical Engineering, St Thomas' Hospital, London SE1 7EH, UK. Email: michelle.ma@kcl.ac.uk; b Department of Chemistry, University College London, 20 Gordon Street, London WC1H 0AJ, UK; c Division of Analytical and Environmental Sciences, King's College London, Franklin Wilkin's Building, London SE1 9NH, UK; d School of Chemistry, Bio21 Molecular Science and Biotechnology Institute, The University of Melbourne, 3010, Victoria, Australia

## Abstract

Gallium-68 (^68^Ga) is a positron-emitting isotope used for clinical PET imaging of peptide receptor expression. ^68^Ga radiopharmaceuticals used in molecular PET imaging consist of disease-targeting biomolecules tethered to chelators that complex ^68^Ga^3+^. Ideally, the chelator will rapidly, quantitatively and stably coordinate ^68^Ga^3+^ at room temperature, near neutral pH and low chelator concentration, allowing for simple routine radiopharmaceutical formulation. Identification of chelators that fulfil these requirements will facilitate development of kit-based ^68^Ga radiopharmaceuticals. Herein the reaction of a range of widely used macrocyclic and acyclic chelators with ^68^Ga^3+^ is reported. Radiochemical yields have been measured under conditions of varying chelator concentrations, pH (3.5 and 6.5) and temperature (25 and 90 °C). These chelators are: 1,4,7,10-tetraazacyclododecane-1,4,7,10-tetraacetic acid (DOTA), 1,4,7-triazacyclononane-1,4,7-triacetic acid (NOTA), 1,4,7-triazacyclononane macrocycles substituted with phosphonic (NOTP) and phosphinic (TRAP) groups at the amine, bis(2-hydroxybenzyl)ethylenediaminediacetic acid (HBED), a tris(hydroxypyridinone) containing three 1,6-dimethyl-3-hydroxypyridin-4-one groups (THP) and the hexadentate tris(hydroxamate) siderophore desferrioxamine-B (DFO). Competition studies have also been undertaken to assess relative complexation efficiencies of each chelator for ^68^Ga^3+^ under different pH and temperature conditions. Performing radiolabelling reactions at pH 6.5, 25 °C and 5–50 μM chelator concentration resulted in near quantitative radiochemical yields for all chelators, except DOTA. Radiochemical yields either decreased or were not substantially improved when the reactions were undertaken at lower pH or at higher temperature, except in the case of DOTA. THP and DFO were the most effective ^68^Ga^3+^ chelators at near-neutral pH and 25 °C, rapidly providing near-quantitative radiochemical yields at very low chelator concentrations. NOTP and HBED were only slightly less effective under these conditions. In competition studies with all other chelators, THP demonstrated highest reactivity for ^68^Ga^3+^ complexation under all conditions. These data point to THP possessing ideal properties for rapid, one-step kit-based syntheses of ^68^Ga-biomolecules for molecular PET imaging. LC-MS and ^1^H, ^13^C{^1^H} and ^71^Ga NMR studies of HBED complexes of Ga^3+^ showed that under the analytical conditions employed in this study, multiple HBED-bound Ga complexes exist. X-ray diffraction data indicated that crystals isolated from these solutions contained octahedral [Ga(HBED)(H_2_O)], with HBED coordinated in a pentadentate N_2_O_3_ mode, with only one phenolic group coordinated to Ga^3+^, and the remaining coordination site occupied by a water molecule.

## Abstract 2

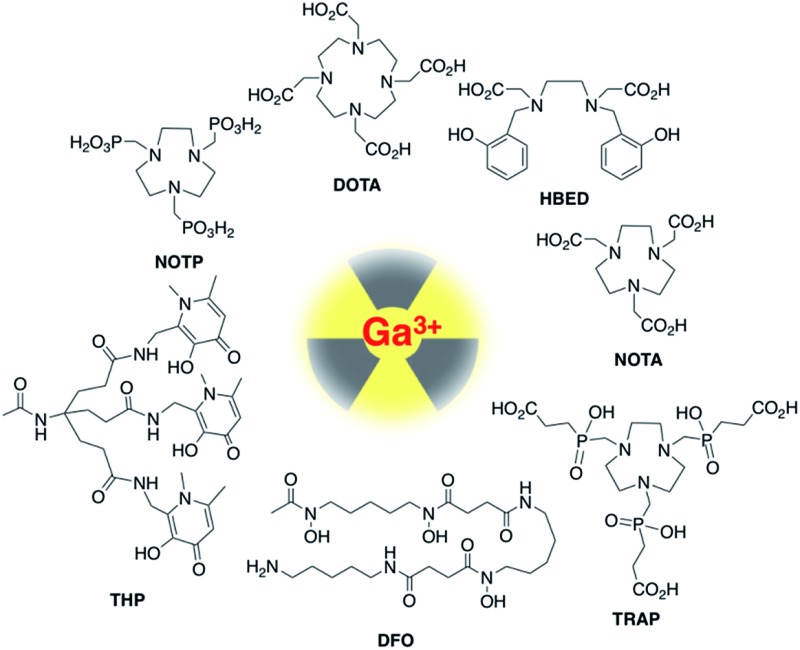
A range of macrocyclic and acyclic chelators have been reacted with the PET isotope, gallium-68, and their radiolabelling efficiencies have been compared. Structural data for complexes of HBED with Ga^3+^ are reported.

## Introduction

Gallium-68 (^68^Ga) is a positron-emitting isotope with emission properties (*t*_1/2_ = 68 min, *β*^+^ 90%, *E*_max_ = 1880 keV) that make it suitable for diagnostic imaging with positron emission tomography (PET). A pharmaceutical grade ^68^Ge/^68^Ga generator has recently become commercially available,^1^ providing hospitals with on-site access to a GMP-grade diagnostic PET radionuclide without the need for local cyclotron facilities. The most widely utilised ^68^Ga radiopharmaceuticals consist of ^68^Ga coordinated to a chelator that is attached to a peptide for targeting cell-surface receptors of tumours. Numerous centres already routinely produce diagnostic ^68^Ga–HBED–PSMA^2^,^3^ and ^68^Ga–DOTA–TATE^4^–^6^ for whole-body PET imaging of prostate and neuroendocrine cancers respectively. These radiotracers have had a significant impact on patient management in centres where they are available, but the complexity of their radiosynthesis in hospitals is a barrier to widespread implementation.

Radiosynthesis of ^68^Ga–DOTA–TATE requires heating at 80–100 °C in order for the DOTA chelator to chelate radiopharmaceutical concentrations of ^68^Ga^3+^ with yields greater than 80%.^7^–^9^ On the other hand, ^68^Ga–HBED–PSMA can be prepared at ambient temperatures, but HBED forms multiple species when complexed to Ga^3+^.^10^ This is undesirable as it is possible that the different species have different pharmacological profiles. Heating is employed to increase formation of the most thermodynamically favoured compound, although the structure of this complex has not been defined. Even with heating, populations of other isomers are observed.^10^ The radiosyntheses of ^68^Ga–DOTA–TATE and ^68^Ga–HBED–PSMA are undertaken at pH 3–5.

As a result of heating requirements at acidic pH, clinical radiosyntheses of both ^68^Ga–DOTA–TATE and ^68^Ga–HBED–PSMA require multiple manipulations or complex automated equipment.^9^,^10^ Typical ^68^Ga radiopharmaceutical syntheses involve (i) elution of ^68^Ga from a generator, (ii) pretreatment of eluate to remove contaminating metal impurities that interfere with radiolabelling, as well as ^68^Ge “breakthrough”, (iii) addition of ^68^Ga to aqueous solutions of peptide–chelator precursor at pH 3–5, (iv) heating for 5–10 min (followed by cooling) (v) removal of unreacted ^68^Ga and buffering salts (using solid phase extraction cartridges) and (vi) reconstitution in physiologically compatible solutions for patient administration. In centres that are equipped for more complex preparations of ^18^F radiopharmaceuticals, this is not a barrier to routine radiosynthesis but it is time-consuming and costly. However, in regional healthcare centres, or hospitals in countries with developing healthcare systems, such complexity will be a barrier to widespread implementation.

**Chart 1:**
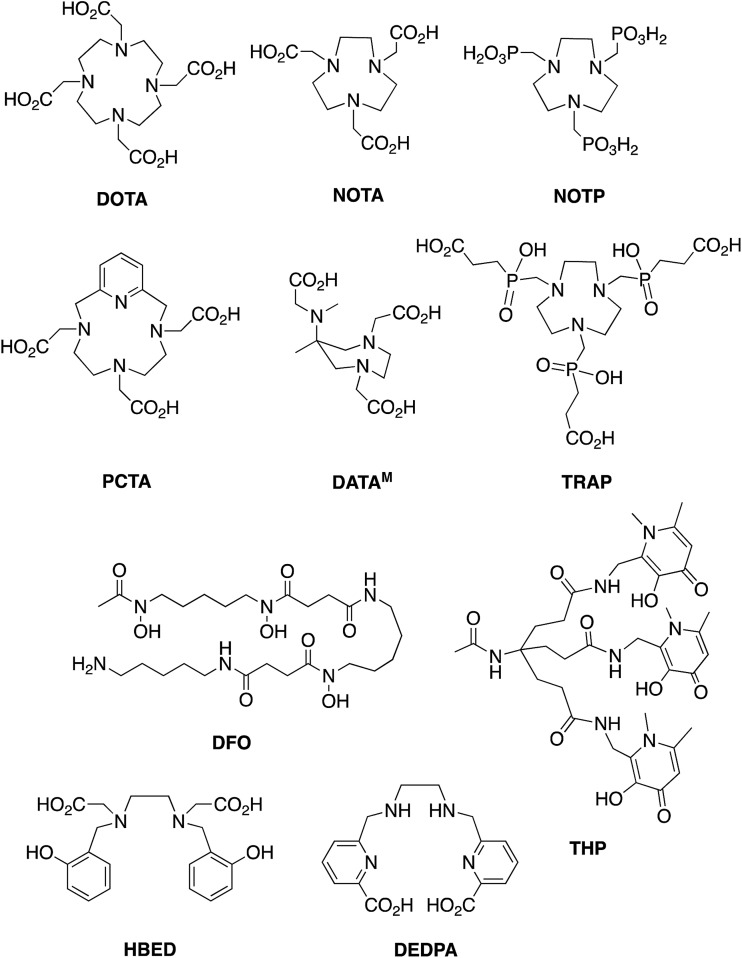


Chelators that quantitatively coordinate ^68^Ga^3+^ at near-neutral pH, room temperature and low concentrations of chelator-bioconjugate will enable one-step, kit-based radiolabelling protocols, with concomitant widespread patient benefit. Such radiosyntheses would ideally only require a kit vial containing bioconjugate and buffer components, ^68^Ga generator eluate, a syringe and appropriate radiation shielding. Chelators that fulfil these requirements would also be useful for radiolabelling of small proteins that are susceptible to unfolding or degradation at extremes of pH and temperature. Over the past decade, several chelators have been evaluated and/or developed for ^68^Ga radiolabelling of biomolecules, to overcome the limitations of DOTA (1,4,7,10-tetraazacyclododecane-1,4,7,10-tetraacetic acid). These include chelators based on 1,4,7-triazacyclononane-1,4,7-triacetic acid (NOTA and its derivative NODAGA),^11^–^15^ 1,4,7-triazacyclononane (tacn) macrocycles substituted with phosphonic (NOTP^16^,^17^) and phosphinic (TRAP^18^,^19^) groups at the amine, hexaazamacrobicycles,^20^ a pyridyl-substituted DOTA macrocycle (PCTA),^15^,^21^ bis(2-hydroxybenzyl)ethylenediaminediacetic acid (HBED) and related compounds possessing phenol, amine and carboxyl donor groups,^3^,^22^ 6-amino-1,4-diazepanes with acetate substituents at the amines (DATA),^23^–^26^ a siderophore-derived macrocyclic chelator with hydroxamate groups (FSC),^27^ the acyclic siderophore desferrioxamine-B (which also contains hydroxamates),^28^,^29^ and an acyclic chelator based on a substituted pyridine carboxylate with an N_4_O_2_ binding mode (DEDPA) (Chart 1).^30^–^32^ Our research group has recently developed tris(hydroxypyridinone) (THP) derivatives based on 1,6-dimethyl-3-hydroxypyridin-4-one units.^33^–^39^ Of these chelators, NOTA/NODAGA, TRAP/NOPO, HBED, FSC, DATA, DFO, DEDPA and THP can reportedly be radiolabelled with ^68^Ga^3+^ at ambient temperature. Only DATA derivatives^24^ and THP derivatives^33^–^35^,^37^,^38^ have been reported to complex ^68^Ga^3+^ above pH 5 at ambient temperature. Many of these ligands provide highly rigid and inert Ga^3+^–chelator complexes. Rigidity is imparted by both selection of appropriate “hard” donor atoms with high affinity for Ga^3+^, which has a relatively high charge density, and the geometry or topology of the chelator itself. Chelators with pre-arranged conformations^18^,^26^,^32^ that accommodate octahedral binding of Ga^3+^ favour very high complex rigidity, contributing to kinetic stability of the resulting Ga^3+^ complex.

Most radiosyntheses of ^68^Ga–chelator complexes have been undertaken in acidic solution, below pH 5. This is because hydrated Ga^3+^ species such as [Ga(H_2_O)_6_]^3+^ predominate in solution below pH 4 but as the pH is raised above 4, the poorly soluble hydroxide species Ga(OH)_3_ is formed, until the pH exceeds 6.3, where tetracoordinate [Ga(OH)_4_]^–^ predominates,^40^–^43^ although this is strongly dependant on temperature and is influenced by concentrations of other metal ions and coordinating molecules in solution.^44^,^45^ For efficient ^68^Ga^3+^ radiolabelling of chelate–peptide conjugates above pH 4, chelate complex formation must effectively compete with ^68^Ga-colloid formation. Preferably, the rate of chelation will be diffusion-controlled, so that complex formation outcompetes ^68^Ga^3+^ colloid formation. The amounts of ^68^Ga eluted from clinical generators are in the range of 400–2000 MBq, approximately equivalent to 4–20 pmol of ^68^Ga^3+^ in 1–5 mL of solution, *i.e.* nanomolar concentrations. Highly efficient chelators are required to quantitatively coordinate such low concentrations of metal ion without excessively high chelator concentrations.

For most of the chelators mentioned above, their Ga^3+^ complexes and their complexes with some other metal ions, metal stability constants and protonation constants have been reported (Table 1). These data are very useful in predicting the ability of a chelator to coordinate Ga^3+^, the selectivity of a chelator for Ga^3+^ over other metal ions, and the ability of other ligands such as hydroxide ions, to compete for Ga^3+^ binding under physiological conditions. These data do not, however, predict the kinetics of complexation, and without very detailed speciation studies, they do not describe the complexity of the reaction matrix in ^68^Ga radiolabelling solutions where other adventitious metal ions are present, as well as buffer components.

**Table 1:** Proton and Ga^3+^ affinity constants of chelators used in this study

Chelator	log *K*_a_	log *K*_1_
DOTA^48^,^49^	11.74, 9.76, 4.68, 4.11, 2.37	26.05
NOTA^18^,^50^	13.17, 5.74, 3.22, 1.96	29.63
NOTP^51^	11.7, 9.1, 7.5, 5.8, 3.1, 0.9	—
TRAP^19^	11.48, 5.44, 4.84, 4.23, 3.45, 1.66	26.24
HBED^52^	12.60, 11.00, 8.44, 4.72, 2.53, 1.74	39.57
DFO^53^	10.79, 9.55, 8.96, 8.32	28.65
Deferiprone^*a*^ ^,^^54^,^55^	9.86, 3.70;^54^ 9.78, 3.61 55	(log *β*_3_) 38.42;^54^ 37.35 55

^*a*^In the absence of published stability constants for THP, we have included log *β*_3_ values for the related [Ga(deferiprone)_3_] complex (deferiprone = 1,2-dimethyl-3-hydroxypyridin-4-one).

To the best of our knowledge, there have been no comprehensive side-by-side comparisons of Ga^3+^ chelators to evaluate relative radiolabelling efficiencies. Several prior studies have compared ^68^Ga radiolabelling for a limited number of chelators.^16^,^21^,^24^,^33^,^35^,^46^,^47^ Some of these studies have compared different chelator concentrations or amounts, and all of these studies only explore one or two specific reaction conditions. In some of these studies including our own, the ^68^Ga radiolabelling conditions used for each chelator are not identical.^33^,^35^,^46^ Therefore, to identify the most suitable chelators to take forward for kit-based ^68^Ga radiolabelling, we have compared the efficiency of ^68^Ga radiolabelling of commercially available DOTA, NOTA, NOTP, TRAP, HBED, THP and DFO, under four different reaction conditions (high and ambient temperatures, and neutral and low pH conditions), and across five orders of magnitude of chelator concentration (50 nM to 500 mM). We also report data that reveal the complexity of Ga^3+^–HBED coordination.

## Experimental

### Materials and instrumentation

All solvents and reagents were obtained from Sigma-Aldrich (Dorset, UK) unless otherwise indicated. DOTA, NOTA and NOTP were purchased from Macrocyclics (Dallas, USA). TRAP was purchased from CheMatech (Dijon, France). HBED was purchased from Santa Cruz Biotechnology (Dallas, USA). DFO was purchased from Sigma-Aldrich. THP was synthesised in our laboratory according to a previously reported procedure.^33^,^56^ The purchased chemicals were used without further purification. NMR spectra were acquired on Bruker Avance 400 MHz spectrometers (either narrow-bore or wide-bore) (Bruker, Germany) equipped with either a 5 mm QNP probe or a 5 mm BBO probe at 298 K. Spectra were referenced to residual solvent signals or TMS. High-performance liquid chromatography (HPLC) analysis was carried out using an Agilent 1200 LC system with in-line UV and gamma detection (Flow-Count, LabLogic). Instant thin layer chromatography plates (iTLC-SG) were obtained from Agilent Technologies (California, USA) and iTLC strips were visualized and quantified using a Cyclone Plus Storage Phosphor System (Perkin Elmer) interfaced with OptiQuant V5.0 software (Perkin Elmer). Analytical reverse phase HPLC were acquired using an Agilent Eclipse XDB-C_18_ column (9.4 × 250 mm, 5 μm) and UV spectroscopic detection at 220 nm. Aliquots (50 μL) of each radiolabelled sample were injected onto the column, using a flow rate of 1 mL min^–1^, and the following gradient: 0–5 min: 100% A/0% B; 5–25 min: 100% A/0% B to 60% A/40% B. Mobile phase A comprised water with 0.1% trifluoroacetic acid and mobile phase B comprised acetonitrile with 0.1% trifluoroacetic acid. Analytical LC-MS were recorded in the positive ion mode on an Agilent 6510 Q-TOF LC/MS mass spectrometer coupled to an Agilent 1200 LC system (Agilent, Palo Alto, CA) and a LabLogic scintillation detector Flow-count system (Sheffield, UK). An Agilent Eclipse XDB-C_18_ column (9.4 × 250 mm, 5 μm) and UV spectroscopic detection at 220 nm was used with a flow rate of 1 mL min^–1^, and the following gradient: 0–5 min: 100% A/0% B; 5–25 min: 100% A/0% B to 0% A/100% B. Mobile phase A comprised water with 0.1% formic acid and mobile phase B comprised acetonitrile with 0.1% formic acid. Elemental analysis was performed by the Science Centre, London Metropolitan University.

### ^68^Ga radiolabelling and iTLC quantification

^68^Ga was eluted from an Eckert & Ziegler ^68^Ge/^68^Ga generator system (Berlin, Germany). Aqueous HCl solution (0.1 M, 5 mL) was passed through the generator and the eluate was collected in 5 × 1 mL fractions. Aliquots of the second fraction (1 mL, containing 130–230 MBq ^68^Ga) were used directly for radiolabelling reactions.

Chelators were dissolved in aqueous solutions of sodium acetate (0.2 M) or ammonium acetate (0.2 M) to provide solutions with chelator concentrations ranging from 50 nM to 1 mM (50 nM, 500 nM, 5 μM, 50 μM, 500 μM, and 1 mM). Ligand solutions were freshly prepared from stock solutions for each experiment. ^68^Ga (10 μL, approx. 2 MBq in 0.1 M aqueous HCl) was added to chelator solutions (100 μL) and the reaction solution was incubated at either 25 or 90 °C. The final pH of the reaction solutions was 3.5 and pH 6.5 for the sodium acetate and ammonium acetate solutions respectively. After 10 min, the reaction solution was analysed by iTLC (glass microfiber chromatography paper impregnated with silica gel, 80 × 10 mm).

Separately, solutions of ^68^Ga^3+^ (10 μL, approx. 2 MBq in 0.1 M aqueous HCl) were added to aqueous solutions of sodium acetate (0.2 M) or ammonium acetate (0.2 M), and incubated at either 25 or 90 °C. After 10 min, the solutions were analysed by iTLC.

Three different mobile phases were employed for iTLC:

(1) For THP, DFO and chelator-free reactions under all reaction conditions, aqueous sodium citrate solution (0.1 M, pH 5.5) was used. [^68^Ga(chelator)] *R*_f_ < 0.1; non-chelated, soluble ^68^Ga^3+^ *R*_f_ > 0.9; ^68^Ga colloids: <0.1. Based on quantification of ^68^Ga colloid and soluble ^68^Ga^3+^ in chelator-free reactions, RCY values were adjusted to account for coincident *R*_f_ values of ^68^Ga colloid, [^68^Ga(THP)] and [^68^Ga(DFO)].

(2) For NOTP under all reaction conditions, and DOTA, NOTA, TRAP and HBED, under all conditions except pH 3.5, 90 °C, aqueous sodium phosphate solution (0.4 M, pH 4) was used. [^68^Ga(NOTP)] *R*_f_ = 0.6–0.7; DOTA, NOTA, TRAP and HBED: [^68^Ga(chelator)] *R*_f_ = 0.8–1; non-chelated ^68^Ga^3+^ *R*_f_ < 0.1.

(3) For DOTA, NOTA, TRAP and HBED at pH 3.5 and 90 °C, an ammonium acetate solution (1 M in 80% methanol, 20% water) was used. [^68^Ga(DOTA)] *R*_f_ = 0.65–0.75; [^68^Ga(NOTA)] *R*_f_ = 0.8–0.9; [^68^Ga(HBED)] *R*_f_ = 0.9–1; [^68^Ga(TRAP)] *R*_f_ = 0.4–0.6; non-chelated ^68^Ga^3+^ *R*_f_ = <0.3. These conditions were selected because after heating ^68^Ga solutions at pH 3.5 and 90 °C, two distinct compounds were observed for non-chelated ^68^Ga^3+^: *R*_f_ = 0–0.1 and 0.7–0.9 using mobile phase (2). Thus, non-chelated ^68^Ga^3+^ could not be distinguished from [^68^Ga(chelator)] using mobile phase (2) after heating at pH 3.5 and 90 °C (except in the case of [^68^Ga(NOTP)]).

iTLC conditions and *R*_f_ values are also summarised in Tables S3–S6.†

iTLC plates were imaged and quantified by digital autoradiography using instruments and software described above.

### ICP-MS analysis of ^68^Ga generator eluate

Fractionated eluate (as described above) was allowed to decay for several days before it was analysed by ICP-MS. The quantification of metal contaminants was carried out on a PerkinElmer NexION 350D Inductively coupled plasma mass spectrometer (ICP-MS) running Syngistix v1.0 software with a CETAC ASX520 autosampler (King's College London, UK). The acquisition mode included 5 replicates averaged to give reported values (Fig. 2, S12 and S13†) for ^27^Al, ^59^Co, ^52^Cr, ^65^Cu, ^56^Fe, ^69^Ga, ^72^Ge, ^55^Mn, ^60^Ni, ^208^Pb, ^45^Sc, ^118^Sn, ^47^Ti, ^51^V, ^66^Zn and ^68^Zn. The dwell time was 50 ms per isotope, with 18 L min^–1^ main argon flow, 1.2 L min^–1^ auxiliary argon flow, 0.97 L min^–1^ nebuliser argon flow (optimised), 1600 W RF power, 0.2 mL min^–1^ sample flow, and KED cell mode with 1.2 mL min^–1^ helium flow.

### HPLC analysis of [^68^Ga(chelator)] complexes and competition studies

^68^Ga^3+^ generator eluate (10 μL in 0.1 M aqueous HCl, approx. 2 MBq) was added to chelator solutions (1 mM chelator, 100 μL in either 0.2 M ammonium acetate, or 0.2 M sodium acetate) and the reaction mixtures were incubated at either 25 °C or 90 °C for 10 min, after which they were applied to an analytical reverse phase C_18_ HPLC column.

For competition studies, ^68^Ga^3+^ generator eluate (20 μL in 0.1 M aqueous HCl, approx. 4 MBq) was added to a solution containing equimolar concentrations of two chelators (each 500 μM in either 200 μL 0.2 M ammonium acetate, or 200 μL 0.2 M sodium acetate) and the reaction mixtures were incubated at either 25 °C or 90 °C for 40 min, after which they were applied to an analytical reverse phase C_18_ HPLC column. Data were processed and analysed using Laura Radiochromatography Software (LabLogic).

### Preparation of [^nat^Ga(HBED)]

A sample of HBED (40 mg, 0.09 mmol) was reacted with Ga(NO_3_)_3_·*x*H_2_O (40 mg, 0.15 mmol, 1.6 equiv.) in aqueous ammonium acetate solution (0.2 M, 5–10 mL) and heated at 90 °C for 30 min. The solution was then applied to an Agilent Eclipse semi-preparative reverse phase XDB-C_18_ column (9.4 × 250 mm, 5 μm) with a 3 mL min^–1^ flow rate, and the reaction solution was purified using a gradient elution, in which mobile phase A consisted of water containing 0.1% TFA and mobile phase B consisted of acetonitrile containing 0.1% TFA. The concentration of B increased at a rate of 1% min^–1^. [Ga(HBED)] eluted with a retention time of 32 min. Fractions containing pure [Ga(HBED)] were combined, lyophilised, and ^1^H and ^13^C{^1^H} NMR spectra (in both D_2_O/CD_3_OD (50%/50%) and D_2_O/CD_3_CN (60%/40%)), and LC-MS chromatograms were acquired. Data are reported in Fig. 4, 5 and S4–S8.†

Crystals of Ga–HBED of were obtained from a solution of D_2_O and CD_3_CN. Anal. calc. for [Ga(HBED)(H_2_O)]·CH_3_CN (C_22_H_26_GaN_3_O_7_): C, 51.39; H, 5.10; N, 8.17. Found: C, 51.23; H, 5.25; N, 8.10. A suitable crystal containing [Ga(HBED)(H_2_O)] was selected and mounted on a nylon loop on a SuperNova Atlas diffractometer with Cu Kα radiation (*λ* = 1.54184 Å). The crystal was kept at 150.4(5) K during data collection. Using Olex2,^57^ the structure was solved with the ShelXS^58^ structure solution program using Direct Methods, and refined with the ShelXL^59^ refinement package using Least Squares minimisation.

### Crystal structure determination

Crystal data for [Ga(HBED)(H_2_O)]·CH_3_CN (C_22_H_26_GaN_3_O_7_) (*M* = 514.18 g mol^–1^): monoclinic, space group *P*2_1_/*c* (no. 14), *a* = 12.96001(14) Å, *b* = 7.01939(10) Å, *c* = 25.0910(3) Å, *β* = 97.7700(10)°, *V* = 2261.60(5) Å^3^, *Z* = 4, *T* = 150.4(5) K, *μ*(CuKα) = 2.093 mm^–1^, *D*_calc_ = 1.510 g cm^–3^, 34 357 reflections measured (6.884° ≤ 2*Θ* ≤ 147.354°), 4541 unique (*R*_int_ = 0.0461, *R*_sigma_ = 0.0263) which were used in all calculations. The final *R*_1_ was 0.0292 (*I* > 2*σ*(*I*)) and w*R*_2_ was 0.0747 (all data). The identification code is xstr0762. Deposit number for [Ga(HBED)(H_2_O)]·CH_3_CN: CCDC ; 1564603.†

## Results

A comprehensive selection of chelators was reacted with generator-produced solutions of ^68^Ga^3+^ at a range of chelator concentrations, at high (90 °C) and room (25 °C) temperatures, and in acidic (pH 3.5) and near neutral (pH 6.5) aqueous acetate solutions. In all cases, the reaction time was 10 min. The chelators are: macrocyclic DOTA, NOTA, NOTP and TRAP, and acyclic HBED, DFO and THP (Chart 1). [Note: for ease of nomenclature, charge and protonation states are not included in these abbreviations of the ligands or complexes.]

### Quantifying the efficiency of ^68^Ga^3+^ chelation

**Fig. 1:**
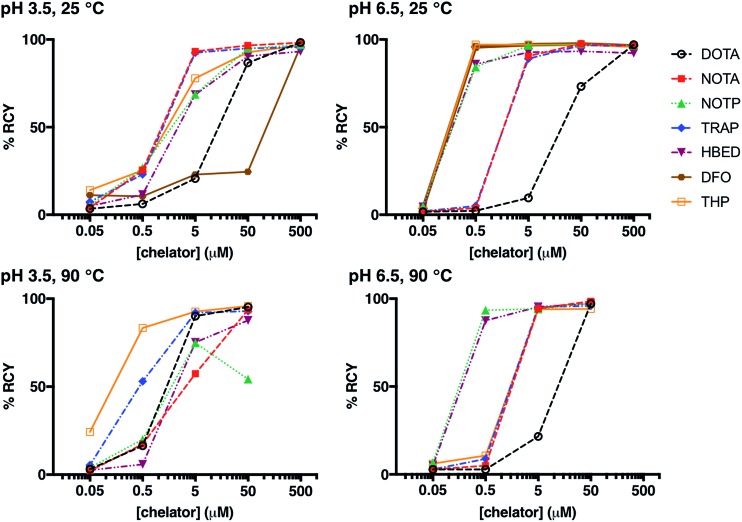
Radiochemical yields for the reaction of ^68^Ga^3+^ with DOTA, NOTA, NOTP, TRAP, HBED, DFO and THP under different concentrations of chelator (500 μM to 50 nM); different pH conditions (pH 3.5 or pH 6.5); and different temperatures (25 °C or 90 °C), after 10 min reaction.

Whilst many ligands will chelate a metal quantitatively if the concentration is high enough, only the most efficient will continue to do so as the concentration is reduced.^12^,^21^,^36^,^46^,^60^,^61^ A series of reactions was undertaken in which a solution of generator-produced ^68^Ga^3+^ (approx. 2 MBq in 0.1 M aqueous HCl, 10 μL) was added to a solution of chelator at a concentration in the range 500 μM to 50 nM (100 μL). The final pH of the reaction solution was 3.5 (using 0.2 M sodium acetate) or 6.5 (using 0.2 M ammonium acetate). After 10 min reaction time, radiochemical yields (RCY) were measured using instant thin layer chromatography (iTLC). Experimental data are supplied for each chelator in Fig. 1, S1, S2† and Table 2. A summary of iTLC conditions is provided in Tables S3–S6.†

**Table 2:** Radiochemical yields (±standard deviation) for the reactions of ^68^Ga^3+^ with DOTA, NOTA, NOTP, TRAP, HBED, DFO and THP. Experiments were undertaken in triplicate

Chelator	Concentration (μM)	pH 3.5, 25 °C	pH 3.5, 90 °C	pH 6.5, 25 °C	pH 6.5, 90 °C
DOTA	500	98.3 ± 0.4		97.0 ± 0.7	
	50	86.7 ± 5.0	95.3 ± 0.9	73.2 ± 6.4	97.2 ± 0.3
	5	20.7 ± 5.6	90.1 ± 1.5	9.6 ± 5.9	21.6 ± 2.5
	0.5	6.2 ± 2.8	16.5 ± 2.2	2.3 ± 0.7	2.8 ± 0.2
	0.05	3.5 ± 1.1	3.1 ± 0.7	1.7 ± 0.5	2.8 ± 0.5
NOTA	500	98.2 ± 0.6		96.6 ± 1.2	
	50	96.2 ± 1.7	93.7 ± 0.3	97.5 ± 0.1	98.5 ± 0.1
	5	93.2 ± 2.0	57.3 ± 2.6	90.6 ± 4.5	94.4 ± 0.4
	0.5	25.4 ± 35.6	17.4 ± 2.1	3.8 ± 0.3	4.9 ± 0.4
	0.05	4.0 ± 2.2	2.2 ± 0.5	1.9 ± 0.7	2.8 ± 0.2
NOTP	500	97.0 ± 0.8		96.7 ± 1.8	
	50	95.0 ± 1.8	54.3 ± 2.4	97.5 ± 0.1	97.3 ± 0.4
	5	68.7 ± 25.0	74.9 ± 3.4	96.6 ± 0.4	94.2 ± 0.9
	0.5	26.8 ± 33.0	20.0 ± 1.9	84.3 ± 0.9	93.5 ± 0.8
	0.05	5.2 ± 3.7	3.9 ± 0.7	4.8 ± 0.4	6.8 ± 1.2
TRAP	500	95.6 ± 0.7		96.5 ± 0.4	
	50	95.0 ± 1.3	93.0 ± 0.6	96.6 ± 0.1	96.0 ± 1.4
	5	92.5 ± 2.4	92.0 ± 1.5	89.0 ± 1.4	95.2 ± 0.4
	0.5	23.0 ± 9.5	53.1 ± 4.8	5.1 ± 0.8	8.8 ± 2.0
	0.05	7.4 ± 1.5	5.5 ± 0.6	2.0 ± 0.5	2.8 ± 0.5
HBED	500	93.2 ± 3.8		92.2 ± 0.6	
	50	90.4 ± 8.1	87.7 ± 0.5	93.3 ± 3.0	97.2 ± 0.4
	5	68.6 ± 23.5	75.2 ± 0.8	92.7 ± 2.2	95.4 ± 0.8
	0.5	11.6 ± 11.9	5.8 ± 0.4	86.0 ± 3.9	87.5 ± 4.4
	0.05	4.9 ± 2.2	2.5 ± 0.4	4.6 ± 0.7	5.1 ± 0.8
DFO	500	96.0 ± 0.8		96.4 ± 1.4	
	50	24.5 ± 1.9		97.5 ± 0.7	
	5	23.0 ± 12.9		97.0 ± 1.0	
	0.5	10.5 ± 5.4		95.8 ± 1.5	
	0.05	11.2 ± 5.0		3.6 ± 1.3	
THP	500	96.7 ± 1.4	96.2 ± 0.8	95.8 ± 1.6	94.3 ± 0.7
	50	92.7 ± 4.2	95.9 ± 0.5	97.1 ± 1.1	94.2 ± 0.6
	5	77.8 ± 13.5	92.7 ± 1.1	97.1 ± 0.1	94.0 ± 0.3
	0.5	25.4 ± 6.8	83.4 ± 1.1	97.1 ± 0.6	10.7 ± 2.6
	0.05	14.0 ± 6.8	24.3 ± 3.4	3.8 ± 3.6	6.1 ± 1.1

#### Room temperature radiolabelling

At pH 3.5, 25 °C, and a chelator concentration of 50 μM, RCYs were greater than 85% for all chelators except DFO (Fig. 1, S1, S2† and Table 2). At 5 μM and 50 μM chelator concentrations, the best performing chelators (*i.e.* that demonstrated highest labelling efficiency) were NOTA and TRAP. At concentrations of 50 μM, RCY of [^68^Ga(NOTA)] was 97 ± 1.7%, and RCY of [^68^Ga(TRAP)] was 95 ± 1.3%. At 5 μM, RCY of [^68^Ga(NOTA)] was 93 ± 2.0%, and RCY of [^68^Ga(TRAP)] was 92 ± 2.4%.

At pH 6.5, 25 °C, and a chelator concentration of 5 μM, RCYs were greater than 85% for all chelators except for DOTA. At pH 6.5 and 25 °C, at very low chelator concentrations of 500 nM and 5 μM, the best performing chelators were DFO and THP. At concentrations of 5 μM, RCY of [^68^Ga(DFO)] was 97 ± 1.0%, and RCY of [^68^Ga(THP)] was 97 ± 0.1%. At 500 nM, RCY of [^68^Ga(DFO)] was 96 ± 1.5% and the RCY of [^68^Ga(THP)] was 97 ± 0.6%.

The p*K*_a_ values for deprotonation of coordinating O donor atoms of NOTA and TRAP are substantially lower than those of DFO and THP (Table 1). At pH 3.5, both NOTA and TRAP complexed ^68^Ga^3+^ with efficiency comparable to that achieved at pH 6.5. At pH 3.5, at concentrations below 500 μM, RCYs of THP and DFO with ^68^Ga^3+^ were relatively poor, however with an increase in pH, Ga^3+^ competed more effectively with protons for coordination to THP and DFO at lower concentrations of chelator. Thus, at pH 6.5, the lowest concentration at which a RCY greater than 95% was reached was 500 nM, achieved using DFO and THP.

#### High temperature radiolabelling

For some macrocycles, the energies of activation for chelation of metal ions are significantly higher than those of linear chelators. To overcome these substantial kinetic barriers when radiolabelling DOTA conjugates with ^68^Ga^3+^, reaction solutions are heated. On the other hand, studies have demonstrated that radioisotopes of Ga^3+^ bind to NOTA and its derivatives at room temperature^12^,^15^ – the kinetic barriers to Ga^3+^ complexation are likely lower for NOTA than DOTA.^11^,^49^ To evaluate the contribution of kinetics to radiolabelling efficiencies at room temperature, radiolabelling reactions (at chelator concentrations of 50 μM to 50 nM) were also undertaken at 90 °C for all chelators except DFO (Fig. 1, S1, S2† and Table 2). HPLC studies (described below) suggested that either DFO or its Ga^3+^ complex decompose at 90 °C.

At pH 3.5, 90 °C, and a chelator concentration of 50 μM, RCYs were greater than 85% for all chelators except NOTP, and the most efficient chelators were THP, DOTA and TRAP. RCY of [^68^Ga(THP)] was 96 ± 0.5%, RCY of [^68^Ga(DOTA)] was 95 ± 0.9% and RCY of [^68^Ga(TRAP)] was 93 ± 0.6%. At 5 μM, RCY of [^68^Ga(THP)] was 93 ± 1.1%, RCY of [^68^Ga(DOTA)] was 90 ± 1.5% and RCY of [^68^Ga(TRAP)] was 92 ± 1.5%. Thus, at pH 3.5, heating substantially improves RCY at chelator concentrations of 500 nM to 5 μM for DOTA and THP. The increased RCY observed for [^68^Ga(DOTA)] at 5 μM at 90 °C (90 ± 1.5%) compared to 25 °C (21 ± 5.6%) is consistent with previous reports.^8^,^9^,^19^ This suggests that the labelling efficiencies of these ligands at pH 3.5 at room temperature are limited by kinetic barriers.

Interestingly, the RCY (57 ± 2.6%) of [^68^Ga(NOTA)] at 5 μM, 90 °C, pH 3.5 was substantially decreased compared to that observed at 25 °C (93 ± 2.0%). It is possible that contaminating metal ions present in generator eluate (see below) effectively compete with Ga^3+^ for NOTA binding at high temperature, but that at lower temperature, the kinetic barriers to complexation of these other metal ions prevent them from competing with Ga^3+^.

At pH 6.5, 90 °C, and a chelator concentration of 5 μM, RCYs were greater than 94% for all chelators except DOTA. In contrast to results observed at room temperature, the best performing chelators were NOTP and HBED. At a chelator concentration of 500 nM, RCY of [^68^Ga(NOTP)] was 94 ± 0.8% and RCY of [^68^Ga(HBED)] was 88 ± 4.4%.

The RCY of [^68^Ga(THP)] at 500 nM and pH 6.5 was substantially reduced at 90 °C (11 ± 2.6%) compared to RCY at 25 °C (97 ± 0.6%). Again, it is possible that THP complexes of metal ion contaminants are formed at high temperature, but not low temperature.

#### ^68^Ga generator ICP-MS eluate analysis

Different batches of ^68^Ga eluate were used for all of the above experiments. Prior work has shown that the concentrations of contaminating metal ions increase with increasing time between ^68^Ga generator elutions.^9^,^62^ In our experiments, the time between elutions was 2–24 hours. To better characterise these reaction solutions, and identify metal ions that compete with ^68^Ga^3+^ for chelator complexation, the concentration of selected metals ions in two batches of generator eluate was quantified, with each eluate fractionated into five samples (each 1 mL). Eluates from two separate elutions were assessed: elution A was obtained 5 hours after the previous elution, and elution B, 150 hours (five days) after the previous elution. The concentrations of Al, Sc, Ti, V, Cr, Mn, Fe, Co, Ni, Cu, Zn (^nat^Zn and ^68^Zn), Ga, Ge, Sn and Pb in generator eluate solution and the hydrochloric acid solution used as eluate were quantified using ICP-MS.

**Fig. 2:**
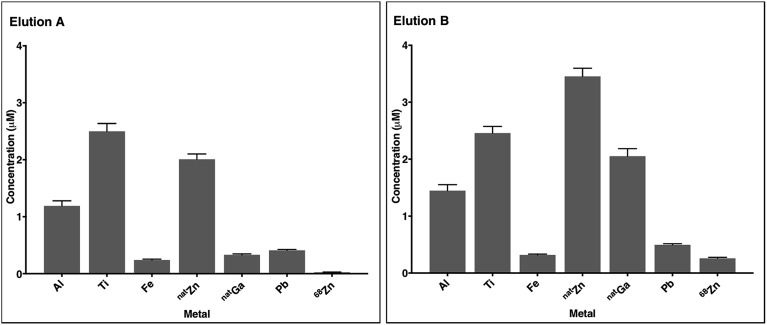
The concentrations of selected metals in ^68^Ga generator eluate from the second 1 mL fraction (measured by ICP-MS). Elution A was obtained 5 hours after the previous elution, and elution B, 150 hours (five days) after the previous elution. Error bars represent standard deviation of the measurement (*n* = 5). ^68^Zn concentrations correspond to ^68^Zn arising from ^68^Ga decay.

In most eluate fractions, including the second fraction used for radiolabelling, Al, Ti, Fe, Zn, Ga and Pb were present at concentrations of 0.1 μM to 5 μM (Fig. 2, S12 and S13†). Concentrations of ^68^Zn (arising from decay of ^68^Ga) were significantly higher in eluate B than eluate A. This is expected: eluate B contained decay products of 150 h of ^68^Ge/^68^Ga decay, whereas eluate A contained decay products of only 5 h of ^68^Ge/^68^Ga decay. Concentrations of ^nat^Ga were also higher in eluate B compared to eluate A. Although the sampling size here is very low, the measured metal contaminant levels fall within a similar range to that of prior reports.^9^ The source of these metal ion contaminants includes the HCl solution used as eluate (contributing a proportion of measured Al, Fe, Zn and Pb), and components of the ^68^Ga generator including titanium dioxide on a borosilicate glass column, lead shielding and tubing.

Complexes of Al^3+^, Ti^4+^, Fe^3+^, Zn^2+^, Ga^3+^ and Pb^2+^ with many of these chelators or their derivatives have been described and characterised. Some of these chelators (for example, HBED,^52^ hydroxypyridinones,^63^ NOTA^18^ and TRAP^18^,^19^) have demonstrated selectivity for Ga^3+^ over divalent ions. However, it is likely that the presence of Al^3+^, Fe^3+^ and ^nat^Ga^3+^, and the high concentrations of Ti^4+^, Zn^2+^ and Pb^2+^, will decrease RCY of the desired ^68^Ga–chelator complex for all chelators used in these experiments. It is also likely that differences in metal concentrations in different eluates leads to variability in RCYs.

### HPLC radiochromatograms of ^68^Ga complexes

Before performing ^68^Ga competition experiments with reverse-phase HPLC, each chelator was reacted with ^68^Ga^3+^ solution, and HPLC radiochromatograms (Fig. S3†) were acquired to determine the retention times and chromatographic behaviour of each complex. For [^68^Ga(DFO)] at 90 °C, multiple signals were observed in the radiochromatograms at both pH 3.5 and 6.5, with wide-ranging retention times. We did not pursue further experiments to elucidate the nature of these species, nor did we study any further reactions of DFO at 90 °C. Others have previously described the structure of [Ga(DFO)].^64^ We postulate that the ligand or complex are not stable at 90 °C, and that the signals correspond to decomposition products.

### Ga–HBED complexes

**Fig. 3:**
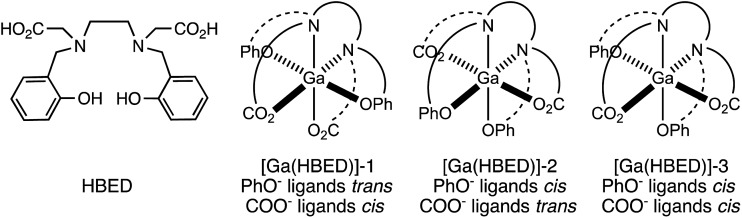
Possible geometric isomers for hexadentate [Ga(HBED)]. Note that each isomer depicted here is one of a pair of enantiomers.

**Fig. 4:**
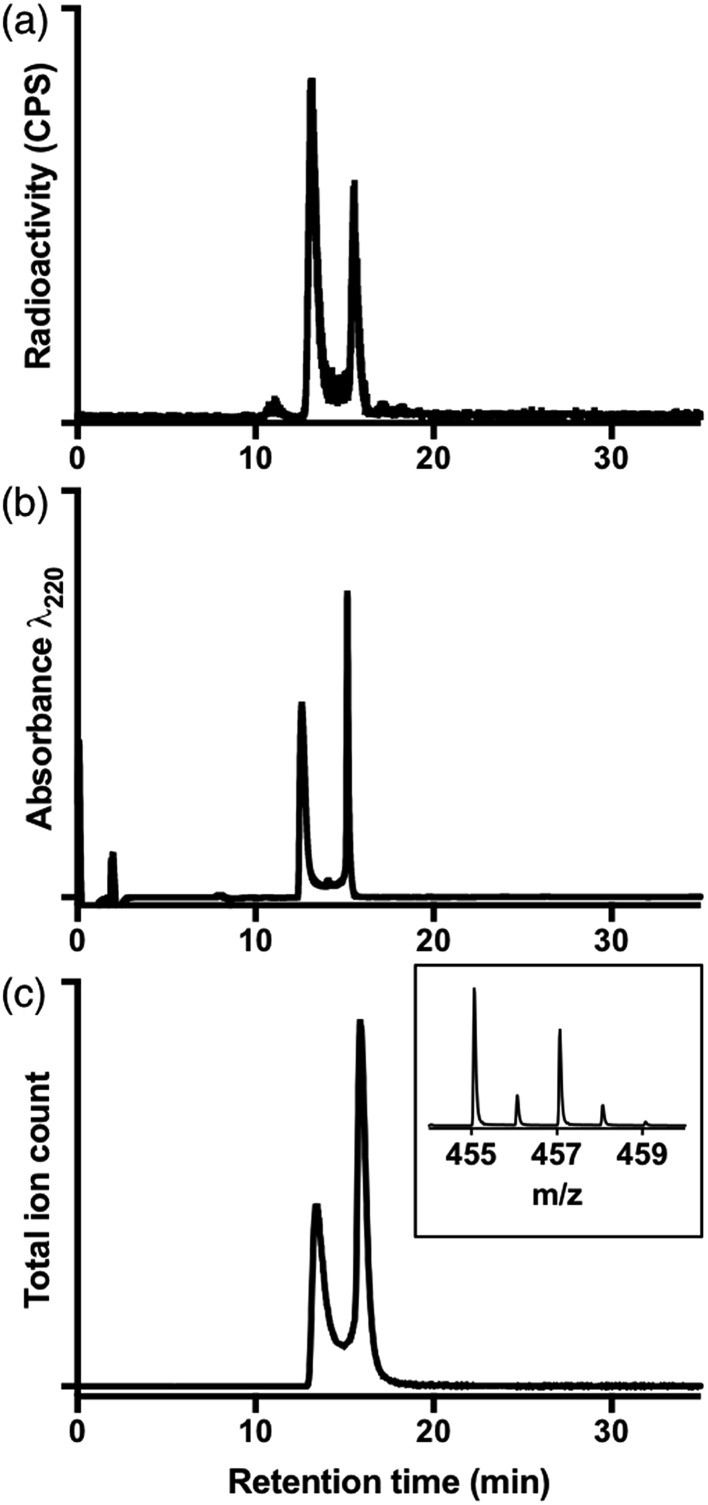
(a) Radiochromatogram of [^68^Ga(HBED)] (prepared at 90 °C); (b) UV chromatogram of [Ga(HBED)]; (c) extracted ion chromatogram of isolated [Ga(HBED)] (*m*/*z* of monoisotopic signal = 455.07); inset positive ion MS spectrum of {[Ga(HBED)] + 2H^+^}: [Ga(C_20_H_22_N_2_O_6_)]^+^.

Prior studies have reported that derivatives of the HBED chelator form isomers when complexed to Ga^3+^ in solution,^10^,^65^ and we have previously suggested that these correspond to geometric isomers (Fig. 3),^66^ however little empirical evidence is available to support this. Consistent with all previous reports of Ga^3+^-bound HBED (Ga–HBED) derivatives, multiple species with distinct HPLC retention times were formed (Fig. 4). At 25 °C, at least three distinct signals in the radiochromatogram of ^68^Ga^3+^–HBED could be distinguished but at 90 °C, only two of these signals were observed (Fig. S4†). The distribution of these species at each temperature was the same whether they were synthesised at pH 3.5 or 6.5.

To further characterise these reaction products, a solution of HBED was reacted with 1.6 equivalents Ga(NO_3_)_3_ at 90 °C, and the resulting complex was isolated (using semi-preparative reverse phase C_18_ HPLC as a mixture of species, including stereo- and possible geometric-isomers). The isolated material was characterised by NMR and LC-MS.

The LC-MS retention times (absorbance at *λ* = 220 nm and total ion count) were coincident with HPLC signals observed from reaction solutions of ^68^Ga^3+^ with HBED at 90 °C (Fig. 4). The product was resolved into two distinct molecular ions by LC-MS, with both corresponding to the expected isotopic pattern for {[Ga(HBED)]^–^ + 2H^+^} (Fig. 4c inset). In the ^13^C{^1^H} spectrum of Ga–HBED, there are four signals corresponding to C=O groups, four signals corresponding to C–O phenolic groups and ten signals (rather than the expected twelve) corresponding to methylene groups. Presumably there are two pairs of coincident signals in the case of the methylene groups. In the ^1^H NMR spectra (including COSY and heteronuclear HSQC ^1^H–^13^C spectra, Fig. S5–S8†), the chemically distinct methylene protons display geminal coupling, and the spectra are also consistent with formation of multiple species. ^13^C{^1^H} and ^1^H spectra were acquired in a mixture of D_2_O/CD_3_CN (60%/40%), and separately, in a mixture of D_2_O/CD_3_OD (50%/50%). Similar spectra were observed for both samples, with no notable differences in the number of signals, nor their chemical shifts and relative intensities. In the ^71^Ga spectrum (acquired in D_2_O/CD_3_CN), a very broad signal is observed, likely arising from overlaid broad resonances corresponding to different Ga–HBED complexes (Fig. 4). A small amount of unchelated Ga^3+^, presumably [Ga(H_2_O)_6_]^3+^, is also present. The broad signal from ^71^Ga reflects the asymmetric environment in all Ga^3+^–HBED species, causing significant quadrupolar relaxation, compared to the symmetric aqua ion.

**Fig. 5:**
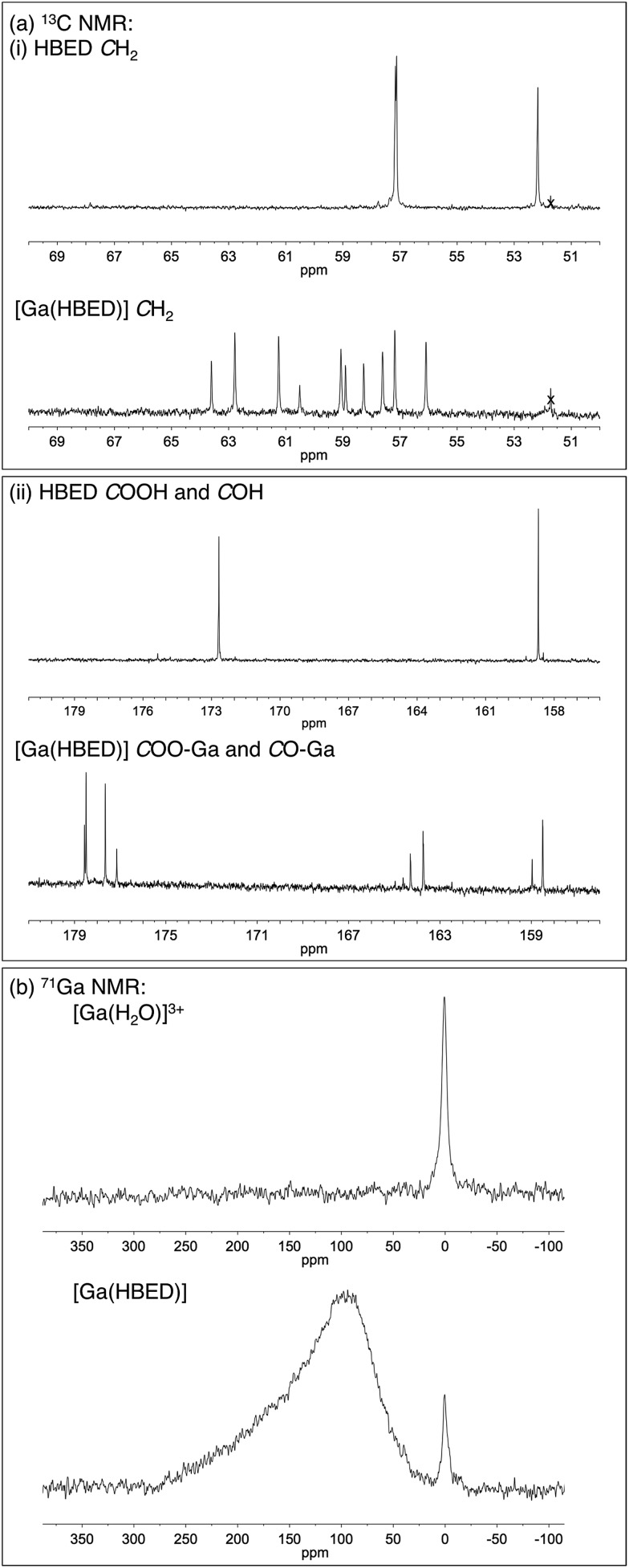
Regions from (a) ^13^C{^1^H} NMR spectra of HBED and [Ga(HBED)], and (b) ^71^Ga NMR spectrum of [Ga(HBED)] (60% D_2_O/40% CD_3_CN). In the ^13^C{^1^H} NMR spectrum of HBED, three *C*H_2_ resonances, one *C*OH resonance and one *C*OOH resonance are detected, but upon coordination to Ga^3+^, an increase in the number of signals is observed. A residual methanol signal is marked (x). In the ^71^Ga NMR spectrum of [Ga(HBED)], a broad, asymmetric peak is observed, distinct from that of unchelated [Ga(H_2_O)_6_]^3+^.

^1^H, ^13^C{^1^H}, COSY, HSQC and ^71^Ga NMR spectra (Fig. 5 and S5–S8†) are consistent with formation of at least two different species. We postulate that these species could include the three possible geometric isomers of a hexadentate N_2_O_4_ species (Fig. 3), as well as complexes in which the HBED ligand coordinates to Ga^3+^ with lower density, (with each species consisting of NMR-indistinguishable Δ and Λ enantiomers). For each of the geometric isomers designated [Ga(HBED)]-1 and [Ga(HBED)]-2, there are three chemically distinct methylene environments, one phenolic environment and one carboxylate environment. For the geometric isomer designated [Ga(HBED)]-3, there are six chemically distinct methylene environments, two phenolic environments and two carboxylate environments. For a Ga–HBED pentadentate species (with a monodentate ligand occupying the sixth coordination site), there are six chemically distinct methylene environments, two phenolic environments and two carboxylate environments.

**Fig. 6:**
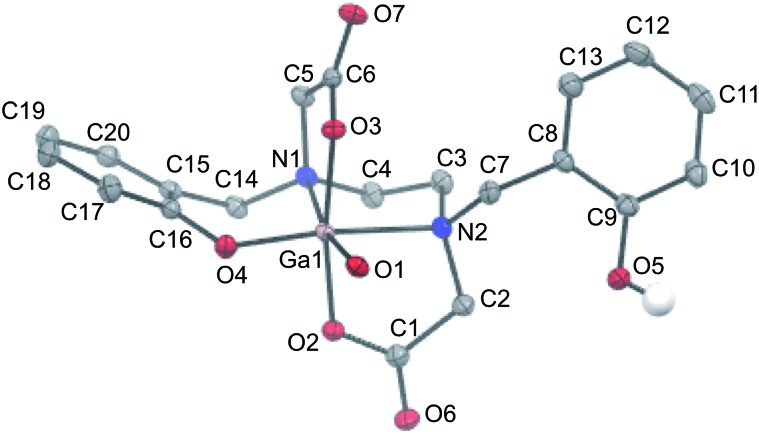
ORTEP representation of [Ga(HBED)(H_2_O)]·CH_3_CN. Ellipsoids are at the 50% probability level. Hydrogen atoms (with the exception of the proton of the non-coordinating phenolic group) and solvent molecules are omitted for clarity.

Slow evaporation of a solution of isolated Ga–HBED material in water and acetonitrile provided crystals suitable for X-ray diffraction (Table S1†). The selected crystal contained the neutral [Ga(HBED)(H_2_O)] complex crystallised with a molecule of acetonitrile, and the unit cell contained four symmetry-related equivalents of [Ga(HBED)(H_2_O)]·CH_3_CN, including both Δ and Λ enantiomers of the Ga^3+^ complex. In [Ga(HBED)(H_2_O)], HBED is bound to Ga^3+^ in a pentadentate N_2_O_3_ environment, with only one phenolic group coordinated to Ga^3+^ (Fig. 6). The non-coordinating phenolic group is protonated and uncharged. A water molecule occupies the remaining Ga^3+^ coordination site to give an octahedral Ga^3+^ complex in which the two carboxylate groups are coordinated *trans* to each other, and the single coordinating phenolic group and H_2_O ligand are *cis* to each other. There is some distortion of the octahedral environment. N–Ga–O bond angles of the five-membered chelate rings, formed by the coordinating carboxylate and amine ligands, are significantly smaller (81.73° and 82.30°) than other bond angles about the metal centre (Table S2†), likely owing to the steric strain in the ligand.

Elemental analysis of the isolated crystalline material indicated that the bulk crystalline material has the same elemental (C, H, N) composition as the single crystal used to acquire X-ray diffraction data (see Experimental section).

Similar geometric arrangements of the HBED ligand have been observed for HBED complexes of Ti^4+^ and Fe^3+^, although in these structures, both phenolic groups are coordinated to the metal centre.^67^,^68^ In hexadentate [Ti(HBED)] and [Fe(HBED)]^–^, the two carboxylate groups are *trans* to each other, and the two phenolic groups are *cis* to each other. The metal–HBED ligand bond lengths in these octahedral complexes are similar to corresponding metal–HBED ligand bond lengths of [Ga(HBED)(H_2_O)].

HPLC chromatograms of solutions of isolated [Ga(HBED)(H_2_O)]·CH_3_CN gave the same peak shapes as previously observed (Fig. S4†).

### Competition studies

**Fig. 7:**
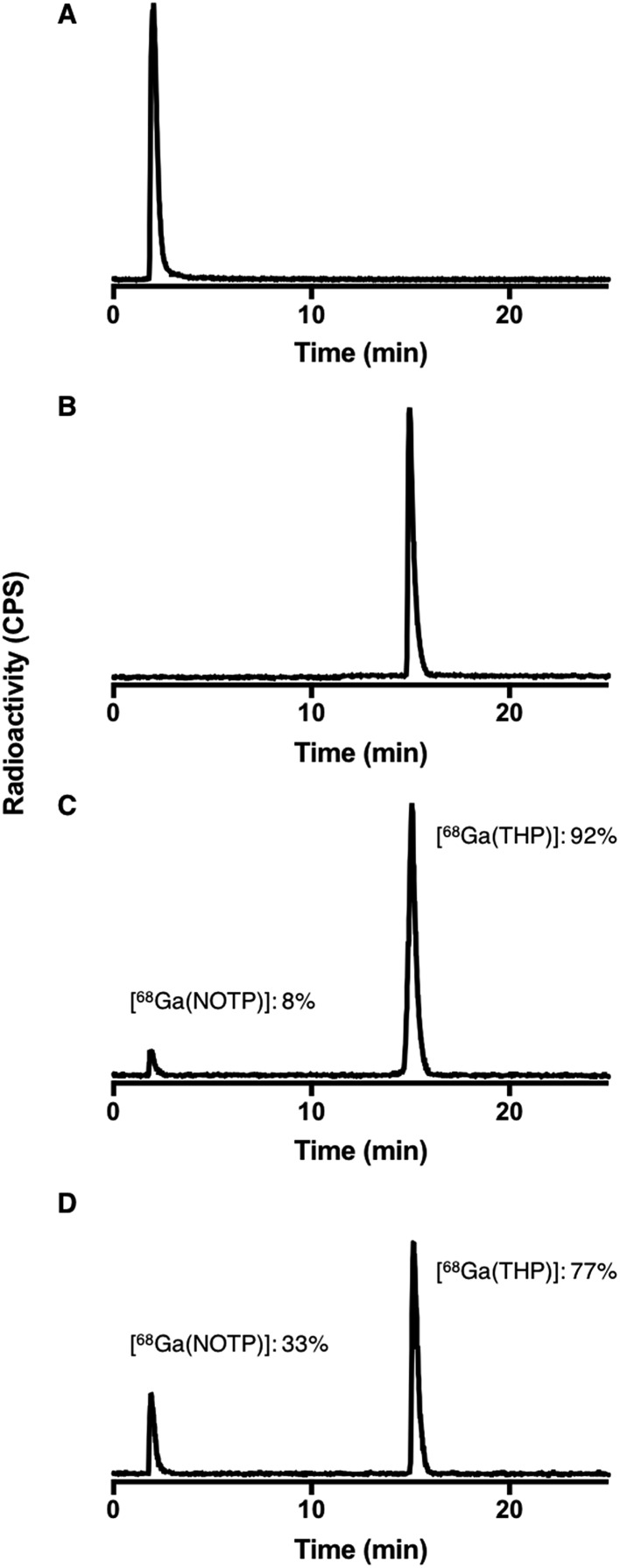
Exemplar HPLC radiochromatograms from competition studies: (A) [^68^Ga(NOTP)] standard; (B) [^68^Ga(THP)] standard; reaction solutions (pH 6.5) containing equimolar concentrations (500 μM) of THP and NOTP with ^68^Ga^3+^ eluate at (C) 25 °C and (D) 90 °C. Radiochromatograms from competition studies are included in ESI, Fig. S9–S11.†

Competition studies^36^ were undertaken, in which two chelators were allowed to compete for ^68^Ga^3+^ binding, at different temperatures (90 °C and 25 °C) and different pH conditions (pH 3.5 and 6.5). Generator eluate containing ^68^Ga^3+^ was added to solutions containing equimolar concentrations of two chelators. The concentration of each chelator was 500 μM. At this concentration, the chelators were in large excess over ^68^Ga^3+^ and the above iTLC studies demonstrated that RCYs for every chelator were near-quantitative. After 40 min reaction (to allow for equilibration), solutions were analysed by reverse phase HPLC (for example, Fig. 7). Radio-chromatographic signals for each ^68^Ga species were integrated, and results summarised as a percentage of total radioactivity (Tables 3 and 4).

**Table 3:** ^68^Ga competition studies at pH 3.5: equimolar solutions of HBED, THP or DFO with either DOTA, NOTA, NOTP, TRAP, HBED, THP or DFO, were reacted with ^68^Ga^3+^. The RCYs of [^68^Ga(HBED)], [^68^Ga(THP)] and [^68^Ga(DFO)] for each of these reactions are given in the below table

	25 °C			90 °C	
	HBED	THP	DFO	HBED	THP
DOTA	99% HBED	100% THP	100% DFO	96% HBED	100% THP
NOTA	43% HBED	97% THP	38% DFO	32% HBED	79% THP
NOTP	0% HBED	94% THP	24% DFO	0% HBED	64% THP
TRAP	0% HBED	100% THP	30% DFO	0% HBED	82% THP
HBED	—	100% THP	17% DFO	—	100% THP
THP	0% HBED	—	0% DFO	0% HBED	—

**Table 4:** ^68^Ga competition studies at pH 6.5: equimolar solutions of HBED, THP or DFO with either DOTA, NOTA, NOTP, TRAP, HBED, THP or DFO, were reacted with ^68^Ga^3+^. The RCYs of [^68^Ga(HBED)], [^68^Ga(THP)] and [^68^Ga(DFO)] for each of these reactions are given in the below table

	25 °C			90 °C	
	HBED	THP	DFO	HBED	THP
DOTA	99% HBED	100% THP	100% DFO	96% HBED	100% THP
NOTA	41% HBED	99% THP	33% DFO	38% HBED	87% THP
NOTP	0% HBED	92% THP	26% DFO	0% HBED	77% THP
TRAP	38% HBED	100% THP	25% DFO	20% HBED	88% THP
HBED	—	100% THP	18% DFO	—	100% THP
THP	0% HBED	—	0% DFO	0% HBED	—

Relative labelling efficiency among DOTA, NOTA, NOTP and TRAP was not compared, as their complexes could not be adequately separated from each other by HPLC or iTLC. ^68^Ga radiolabelled complexes of HBED, DFO and THP showed distinct retention times under the HPLC conditions employed, and labelling efficiencies of these chelators could be compared with each other and with each of DOTA, NOTA, NOTP and TRAP. These competitive comparisons were repeated at pH 3.5 and 6.5, and 25 and 90 °C, except for those involving DFO at 90 °C.

Under all conditions, THP “competed” most effectively for ^68^Ga^3+^ in comparison with all other chelators (Fig. S9†). In each competition study involving THP at 25 °C, RCY of [^68^Ga(THP)] was in the range 92–100%. At 90 °C, the proportion of ^68^Ga^3+^ complexed by the three triazacyclononane (tacn) derivatives (NOTA, NOTP and TRAP) in competition with THP was significantly higher than at 25 °C, although RCYs of [^68^Ga(THP)] still exceeded 60% in these reactions. This suggests that, to some extent, these ^68^Ga reaction products at 25 °C are a result of kinetic preferences.

For competition reactions between THP and DOTA, and between THP and HBED, RCYs of [^68^Ga(THP)] were 100% under all conditions.

The three tacn derivatives competed favourably for ^68^Ga^3+^ in reactions with either HBED or DFO (Fig. S10 and S11†). Indeed in all such competitive reactions, RCYs of ^68^Ga with either NOTA, NOTP or TRAP exceeded 55%. Generally, of the three tacn derivatives, radiochemical yields of [^68^Ga(NOTP)] were highest across all competition studies.

Under the tested conditions, HBED “outcompeted” only DOTA and DFO (Fig. S9 and S10†). At 25 °C under both pH conditions, DFO “outcompeted” only DOTA (Fig. S9–S11†). Compared to all other chelators and under all tested conditions, DOTA was least able to compete for ^68^Ga^3+^ complexation.

There were no remarkable differences between competition studies undertaken at pH 3.5 and pH 6.5, except in the case of reactions of ^68^Ga^3+^ with HBED and TRAP (Fig. S10†). At pH 3.5, all ^68^Ga^3+^ was bound to the TRAP chelator, whereas at pH 6.5, only 60–80% of added ^68^Ga^3+^ was bound to TRAP (depending on the temperature).

## Discussion

There is a prevailing notion in the radiochemical literature, based largely on knowledge of the pH-dependence of the hydrolytic behaviour of the Ga^3+^ aqua ion in forming relatively insoluble hydroxides, that ^68/67^Ga^3+^ chelation is most effective at pH 5 or lower. Most reactions that assess ^68/67^Ga complexation are undertaken at low pH values. Our results demonstrate that for ^68^Ga-radiolabelling of NOTA, NOTP, TRAP, HBED, DFO and THP at 25 °C under these specific reaction conditions, RCYs at pH 6.5 are equal to or greater than RCYs achieved at pH 3.5.

Many reported ^68^Ga radiolabelling experiments have been conducted at temperatures greater than 50 °C. Our results demonstrate that for the majority of chelators, heating the reaction does not significantly increase RCY. The exceptions to this are reactions of ^68^Ga^3+^ with DOTA at both pH 3.5 and pH 6.5, and THP at pH 3.5. In our hands, heating and low pH conditions are only favourable in the case of DOTA – the chelator that is currently used the most for clinical ^68^Ga biomolecule labelling. Even under low pH and high temperature conditions, near-quantitative RCYs for DOTA labelling (greater than 95%) were only achieved at concentrations of 50 μM and above. In these studies, in which low amounts of ^68^Ga were used, this corresponded to maximum specific activities of 20–40 MBq μmol^–1^.

In contrast, near-quantitative RCYs were achieved for THP and DFO at pH 6.5 and 25 °C, at chelator concentrations as low as 500 nM, and in aqueous solutions (ammonium acetate solution) that are physiologically compatible. This corresponded to specific activities of approximately 2–4 GBq μmol^–1^. For reactions that achieved near quantitative RCYs, the maximum specific activity for [^68^Ga(THP)] and [^68^Ga(DFO)] (under mild conditions) was two orders of magnitude higher than that achieved for [^68^Ga(DOTA)] (under low pH and high temperature conditions). High radiochemical yields (>80%) were achieved for NOTP and HBED under the same mild ^68^Ga-labelling conditions. Identifying chelators such as these, that enable reproducible and near-quantitative ^68^Ga biomolecular labelling under low chelator concentration, mild conditions and in physiologically compatible solutions will facilitate (i) one-step, kit-based radiosynthesis of ^68^Ga radiopharmaceuticals; and (ii) ^68^Ga radiolabelling of small proteins (<50 kDa). Proteins that accumulate at target tissue and clear circulation in less than four hours (including engineered antibody derivatives and recombinant proteins) have utility in imaging *in vivo* receptor expression,^47^,^69^–^71^ but many are likely to be sensitive to extremes of pH and temperature.

^67^Ga-labelled DFO–protein conjugates have previously demonstrated *in vivo* and *in vitro* instability, with DFO releasing ^67^Ga^3+^ (between 20–60% dissociation of ^67^Ga^3+^ to serum proteins over three days in solutions containing serum proteins).^28^ This is likely a result of kinetic instability of the [Ga(DFO)] complex, leading to transmetallation of Ga^3+^ to endogenous ligands (proteins, peptides and bone mineral) *in vivo* and *in vitro*. However, the shorter half-life of ^68^Ga renders prolonged *in vivo* stability unnecessary. As DFO complexes ^68^Ga^3+^ in near quantitative yields under mild conditions at low concentrations, DFO is possibly very useful for molecular imaging with ^68^Ga.^72^

At pH 6.5 and 25 °C, NOTP is more efficient at chelating ^68^Ga^3+^ than either of the other tacn derivatives, NOTA and TRAP, and the radiochemical yield for [^68^Ga(NOTP)] is greater than 95% at chelator concentrations of 5 μM. This corresponded to specific activities of 200–400 MBq μmol^–1^. NOTP is also potentially very useful for ^68^Ga biomolecule labelling under mild conditions. Additionally, out of all chelators except THP, NOTP competes most effectively for ^68^Ga^3+^ in competition reactions. Whilst other tacn derivatives, TRAP and NOTA, have been extensively studied for ^68^Ga biomolecule labelling, ^68^Ga bioconjugates of NOTP have not been reported. Reassessment of its utility would be timely.

At pH 6.5 at both high and low temperatures, HBED efficiently chelates ^68^Ga^3+^, achieving 85% RCY at concentrations as low as 500 nM. LC-MS and NMR data for HBED complexes of ^68/nat^Ga^3+^ indicate that under the analytical conditions described here, more than one chemically distinct species exists in solution. It is possible that neutral pentadentate complexes, such as [Ga(HBED)(H_2_O)] observed by single crystal X-ray diffraction (Fig. 6), exist within this population of Ga–HBED species. In light of the geometric arrangement of donor atoms in crystals of [Ga(HBED)(H_2_O)]·CH_3_CN, and existing data on hexadentate HBED complexes of Ti^4+^ and Fe^3+^,^67^,^68^ we postulate that hexadentate [Ga(HBED)]-2 (Fig. 3) is also present in solution.

The analytical chromatographic and spectroscopic conditions used in this study (and in prior studies^10^,^65^) do not mimic physiological conditions. Nonetheless, the data demonstrate the intricate speciation of HBED complexes of Ga^3+^. Detailed speciation studies under physiologically-relevant conditions are required. The log *K*_a_ values for phenolic protons of HBED are 12.6 and 11.0,^52^ and it is possible that the different species of Ga–HBED (and indeed Ga–HBED–PSMA) arise from fluxionality in the coordination/dissociation of the phenolic oxygen ligands. It is likely that in solutions at higher pH values than those studied here, Ga–HBED exists exclusively as a hexadentate complex. It is also possible that a hexadentate complex would be observed in the solid state, if proton counter ions were substituted for other cations.

Competition studies, in which an equimolar solution of two chelators was reacted with ^68^Ga^3+^, indicated that out of all tested chelators and under all tested conditions (which were not concentration-limited), THP competed most effectively for Ga^3+^. These data, alongside THP radiolabelling studies that demonstrate very high ^68^Ga radiolabelling efficiency, point to THP possessing ideal properties for rapid radiolabelling under mild conditions.^33^–^35^,^37^,^39^ It is likely that other chelators that have demonstrated suitable properties for kit-based radiolabelling, such as DEDPA,^30^–^32^ FSC,^27^ and DATA,^24^,^25^ which we have not tested in this study, possess similar properties. Such properties will enable rapid, one-step kit-based syntheses of ^68^Ga-biomolecules for molecular PET imaging without the requirement for complex automated equipment or specialist radiochemistry expertise. This will be key to providing many more hospitals and patients with access to ^68^Ga radiopharmaceuticals.

Whilst thermodynamic and kinetic studies can predict the utility of a chelator for binding very low concentrations of metal ions, it is difficult to compile all necessary data to reliably model the complex reaction matrix of radiolabelling solutions. These solutions contain adventitious metal ions present in concentrations exceeding that of ^68^Ga, and buffer/salt components, which can coordinate both Ga^3+^ and other metal ions, and determine metal ion speciation and reactivity. The simple radiolabelling experiments that we have described here enable identification of suitable and efficient chelators for kit-based ^68^Ga^3+^ radiolabelling, in a reaction matrix typical of radiopharmaceutical formulations.

## Author contributions

M. I. T. and M. T. M. performed radiolabelling experiments, C. E. K. performed crystallography, C. A. F. and M. T. M. synthesised HBED complexes of Ga(iii), C. R. M. and A. C. performed ICP-MS analyses, C. I., J. D. Y. and B. M. P. helped to devise experiments and co-authored the manuscript, T. R. E. performed NMR analysis, P. J. B. and M. T. M. conceived of this study and authored the manuscript.

## Conflicts of interest

P. J. B. is a named inventor on related patents. All other authors have no conflicts to declare.

## Supplementary Material

Supplementary information

Crystal structure data
