# Supplementary material for: Comparison of macrocyclic and acyclic chelators for gallium-68 radiolabelling
Source: RSC Adv. 2017 Oct 25;7(78):49586–99. doi: 10.1039/c7ra09076e (PMC5708347; doi:10.1039/c7ra09076e)
Supplement: Supplementary file 1 [file RA-007-C7RA09076E-s001.pdf]

Electronic Supplementary Material (ESI) for RSC Advances.
This journal is © The Royal Society of Chemistry 2017

Supporting information:

Comparison of macrocyclic and acyclic chelators for gallium-68 radiolabelling

Maria Iris Tsionou,[a] Caroline E. Knapp,[b] Calum A. Foley,[a] Catherine R. Munteanu,[a] Andrew Cakebread,[c] Cinzia Imberti,[a] Thomas R. Eykyn,[a] Jennifer D. Young,[a] Brett M. Paterson,[d] Philip J. Blower,[a] Michelle T. Ma[a]*

* Corresponding author: michelle.ma@kcl.ac.uk

[a] King's College London, Division of Imaging Sciences and Biomedical Engineering, St Thomas' Hospital, London SE1 7EH, UK.

[b] Department of Chemistry, University College London, 20 Gordon Street, London WC1H 0AJ, UK

[c] Division of Analytical and Environmental Sciences Division, Franklin Wilkin's Building, King's College London, London SE1 9NH, UK

[d] School of Chemistry and Bio21 Molecular Science and Biotechnology Institute, The University of Melbourne, Melbourne, Australia

Figure S1. Radiochemical yields for the reaction of $^{68}Ga^{3+}$ with macrocyclic chelators DOTA, NOTA, NOTP and TRAP Page 2

Figure S2. Radiochemical yields for the reaction of $^{68}Ga^{3+}$ with acyclic chelators HBED, DFO and THP Page 3

Figure S3. Reverse phase C_{18} HPLC chromatograms of [^{68}Ga(chelator)] complexes Page 4

Figure S4. Reverse phase C_{18} HPLC chromatograms of [Ga(HBED) Page 4

Figure S5. ^{1}H NMR spectra of HBED and [Ga(HBED)] Page 5

Figure S6. ^{13}C{^{1}H} NMR spectra of HBED and [Ga(HBED)] Page 5

Figure S7. ^{13}C{^{1}H} NMR spectra of HBED and [Ga(HBED)] Page 6

Figure S8. HSQC NMR spectrum of HBED and [Ga(HBED)] Page 6

Figure S9. Exemplar reverse phase C_{18} HPLC chromatograms of solutions from $^{68}Ga^{3+}$ competition studies between THP and other chelators at 25 °C Page 7

Figure S10. Exemplar reverse phase C_{18} HPLC chromatograms of solutions from $^{68}Ga^{3+}$ competition studies between HBED and other chelators at 25 °C Page 7

Figure S11. Exemplar reverse phase C_{18} HPLC chromatograms of solutions from $^{68}Ga^{3+}$ competition studies between DFO and other chelators at 25 °C Page 8

Figure S12. Concentrations of selected metals in ^{68}Ga generator eluate solution Page 8

Figure S13. Concentrations of all analysed metals in ^{68}Ga generator eluate solution Page 9

Table S1. Crystallographic data Page 9

Table S2. Selected bond lengths and angles of [Ga(HBED)(H_2O)].CH_3CN Page 10

Table S3. Mobile phase conditions for iTLC analyses Page 10

Table S4. R_f values of [^{68}Ga(THP)], [^{68}Ga(DFO)] and unreacted ^{68}Ga in mobile phase 1 Page 10

Table S5. R_f values of [^{68}Ga(DOTA)], [^{68}Ga(NOTA)], [^{68}Ga(NOTP)], [^{68}Ga(TRAP)] and [^{68}Ga(HBED)] and unreacted ^{68}Ga in mobile phase 2 Page 11

Table S6. R_f values of [^{68}Ga(DOTA)], [^{68}Ga(NOTA)], [^{68}Ga(NOTP)], [^{68}Ga(TRAP)] and [^{68}Ga(HBED)] and unreacted ^{68}Ga in mobile phase 3 (for reactions undertaken at pH 3.5, 90 °C) Page 11

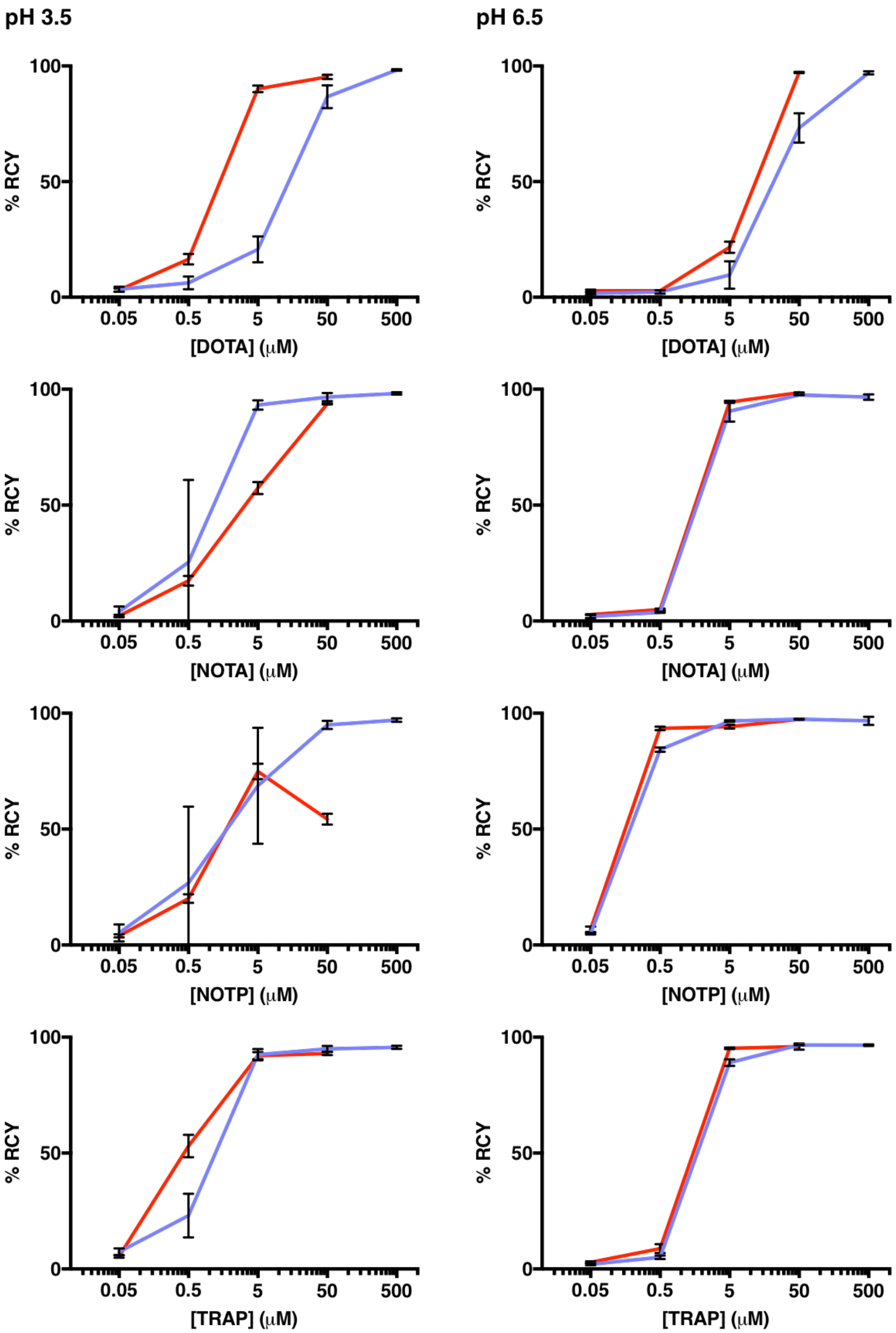

Figure S1. Radiochemical yields for the reaction of $^{68}Ga^{3+}$ with macrocyclic chelators DOTA, NOTA, NOTP and TRAP under different concentrations of chelator (500 μM – 50 nM); different pH conditions (*left* pH 3.5, *right* pH 6.5); and different temperatures (*blue* 25 °C, *red* 90 °C). In all cases, reactions were incubated for 10 min. Experiments were undertaken in triplicate, and error bars correspond to standard deviation.

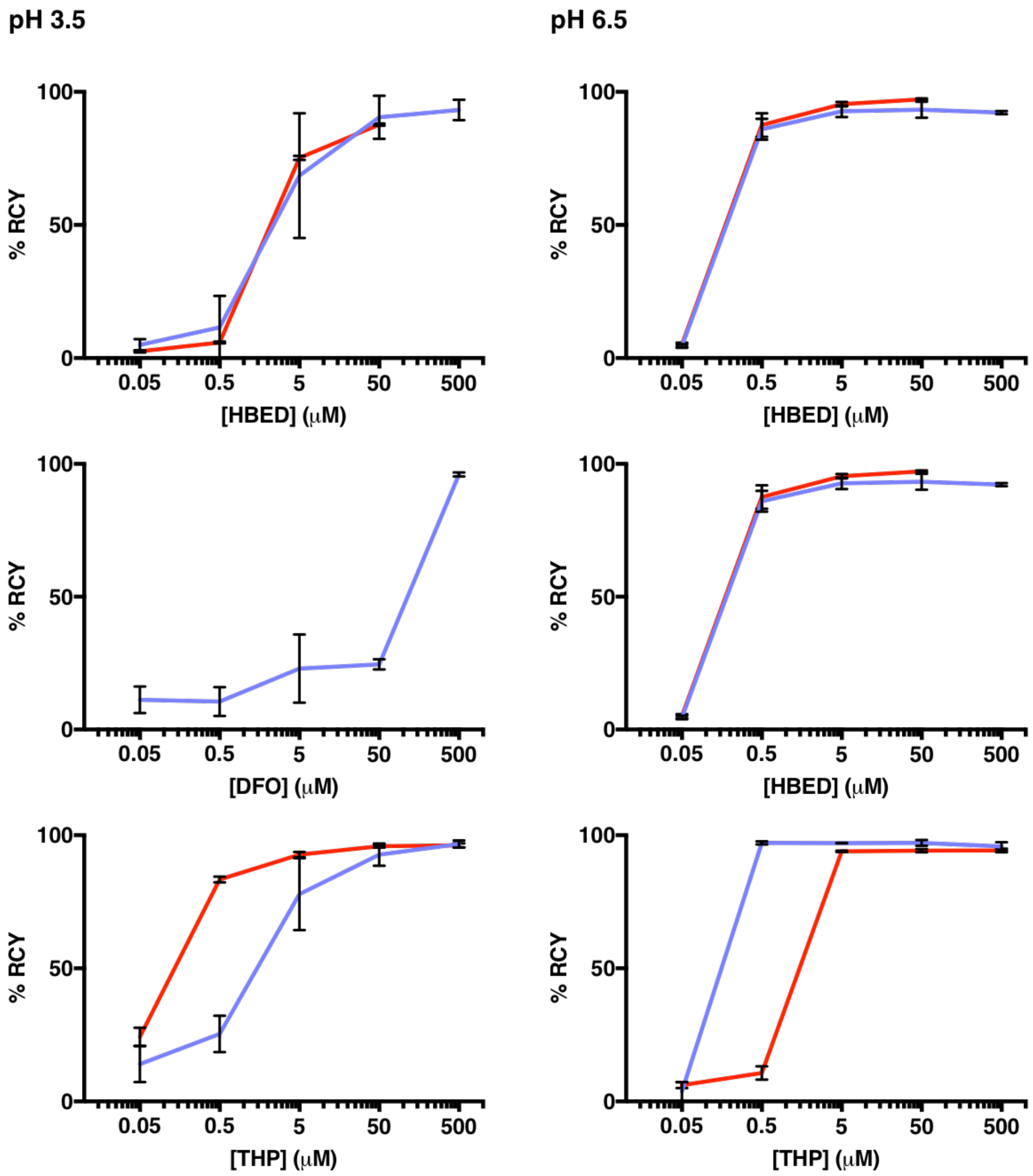

Figure S2. Radiochemical yields for the reaction of $^{68}Ga^{3+}$ with acyclic chelators HBED, DFO and THP under different concentrations of chelator (500 μM – 50 nM); different pH conditions (*left* pH 3.5, *right* pH 6.5); and different temperatures (*blue* 25 °C, *red* 90 °C). In all cases, reactions were incubated for 10 min. Experiments were undertaken in triplicate, and error bars correspond to standard deviation.

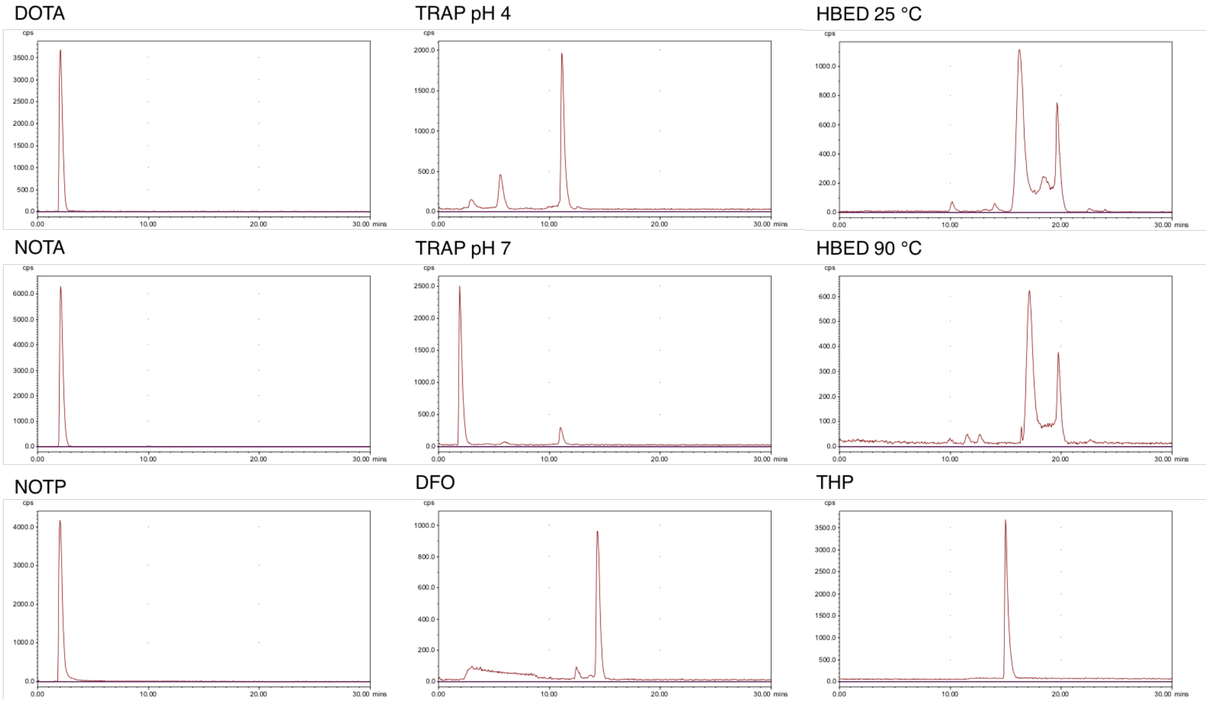

Figure S3. Reverse phase C_{18} HPLC radiochromatograms of [^{68}Ga(chelator)] complexes. Multiple radioactive signals were observed in radiochromatograms of **[^{68}Ga(TRAP)]**. The physicochemical properties of [Ga(TRAP)] have previously been extensively characterised: TRAP reacts rapidly with Ga^{3+} to form an N_3O_3 complex between pH 1.5 and 8, with no reaction intermediates detected, and the complex is stable to acid dissociation over periods of weeks to months. (J. Notni, P. Hermann, J. Havlickova, J. Kotek, V. Kubicek, J. Plutnar, N. Loktionova, J. Riss Patrick, F. Rosch and I. Lukes, *Chem. Eur. J.*, 2010, **16**, 7174-7185.) The pK_a values for [Ga(TRAP)] are 0.7, 3.8, 4.5 and 5.2. It is likely that multiple signals in the radiochromatogram of [^{68}Ga(TRAP)] arise from the separation and detection of different protonation states of the complex in the acidic mobile phase containing 0.1% trifluoroacetic acid. The relative proportion of each species is dependent on the pH of the solution applied to the column, but not the temperature of the reaction. In the case of **[^{68}Ga(HBED)]**, at 25 °C at least three distinct signals in the radiochromatogram of [^{68}Ga(HBED)] can be distinguished but at 90 °C, only two distinct signals are observed in this region of the radiochromatogram.

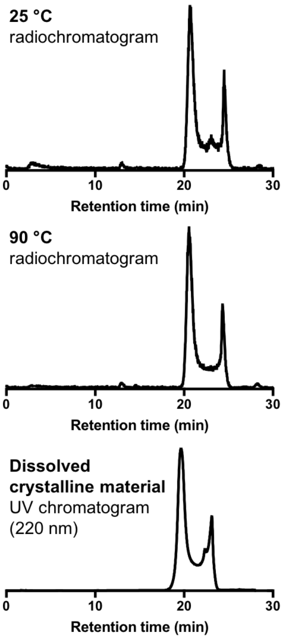

Figure S4. Reverse phase C_{18} HPLC radiochromatograms of **[^{68}Ga(HBED)]** after radiolabelling at 25 °C (top) and 90 °C (middle). At 25 °C at least three distinct signals can be distinguished but at 90 °C, only two distinct signals are observed. UV chromatogram (bottom) of a solution of dissolved crystalline [Ga(HBED)(H_2O)].CH_3CN.

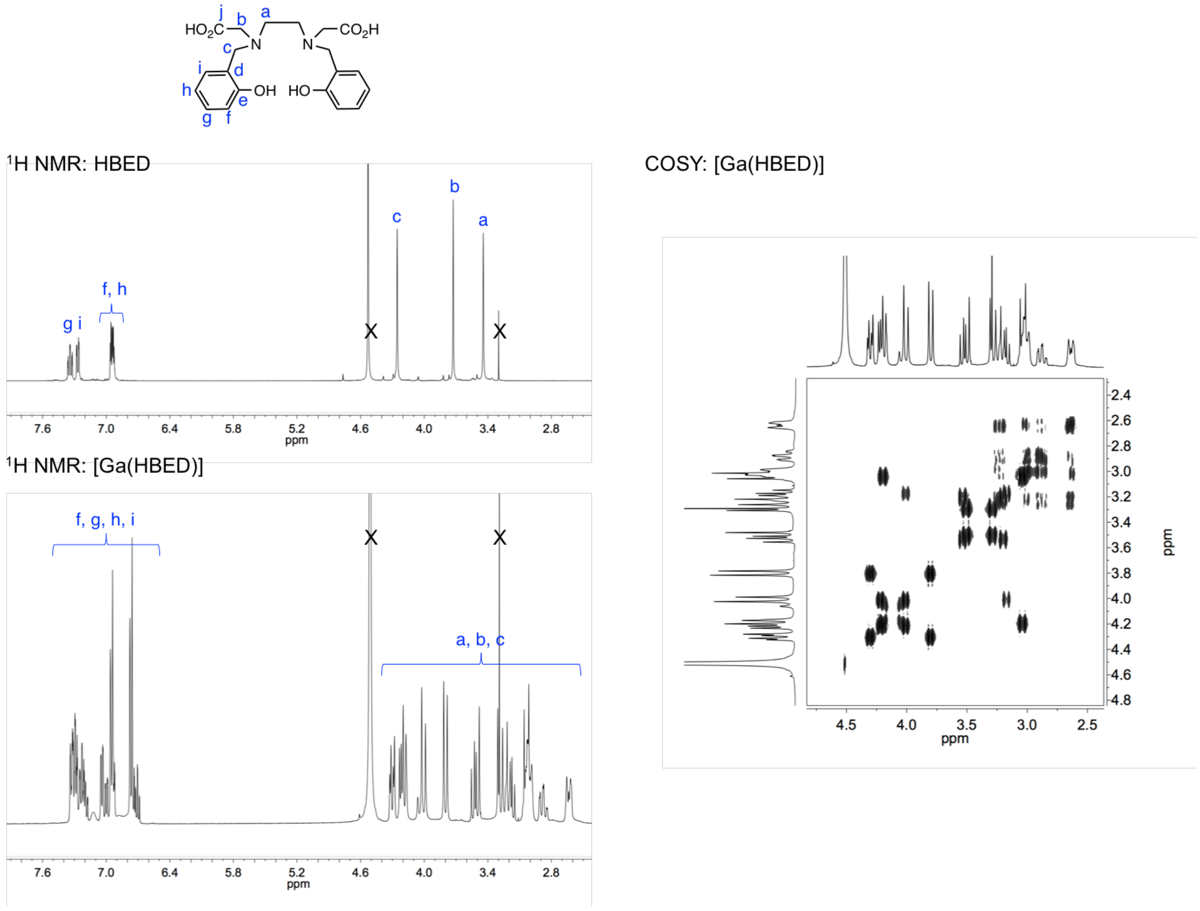

Figure S5. ${}^{1}H$ NMR spectra of HBED (top) and [Ga(HBED)] (bottom), and COSY spectrum of [Ga(HBED)] (right), in 60% D_2O / 40% CD_3CN. ${}^{1}H$ NMR and COSY spectra of [Ga(HBED)] are consistent with the formation of multiple species.

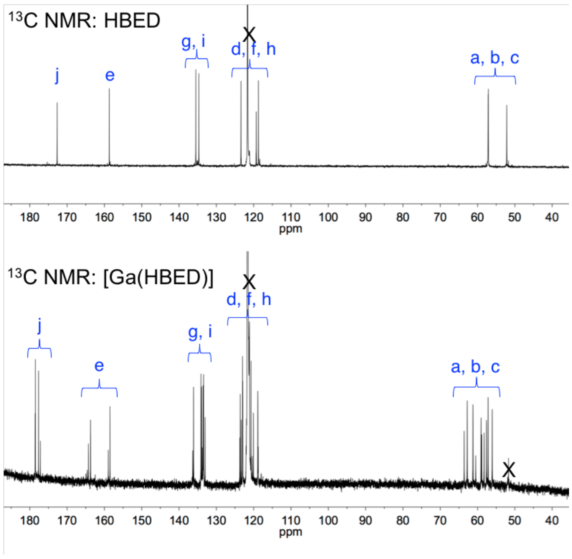

Figure S6. ${}^{13}C\{{}^{1}H\}$ NMR spectra of HBED (top) and [Ga(HBED)] (bottom), in 60% D_2O / 40% CD_3CN. The ${}^{13}C\{{}^{1}H\}$ spectrum of [Ga(HBED)] is consistent with the formation of multiple species. The number of species could not be unequivocally defined based on the existing data, because of the significant amount of spectra overlap of signals in both ${}^{1}H$ and ${}^{13}C\{{}^{1}H\}$ NMR spectra.

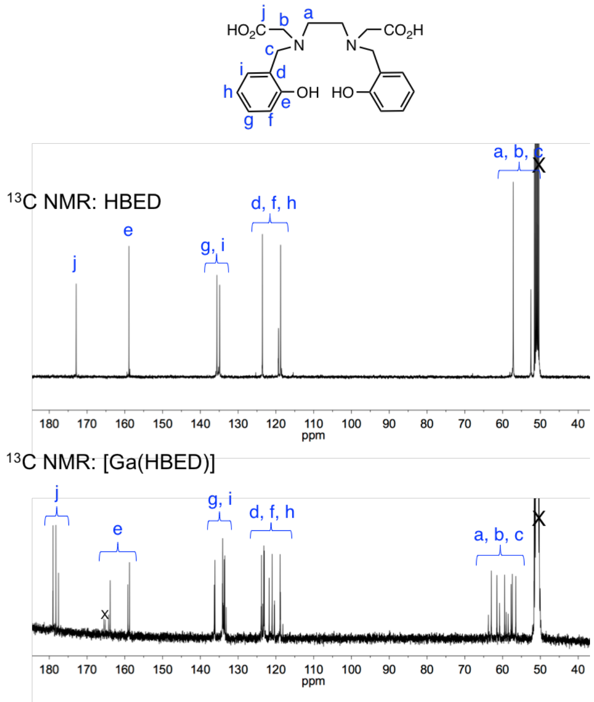

Figure S7. $^{13}C\{^{1}H\}$ NMR spectra of HBED (top) and [Ga(HBED)] (bottom), in 50% D_2O / 50% CD_3OH. In these spectra, signals from the aromatic region do not overlap with solvent signals (as in Figure S6).

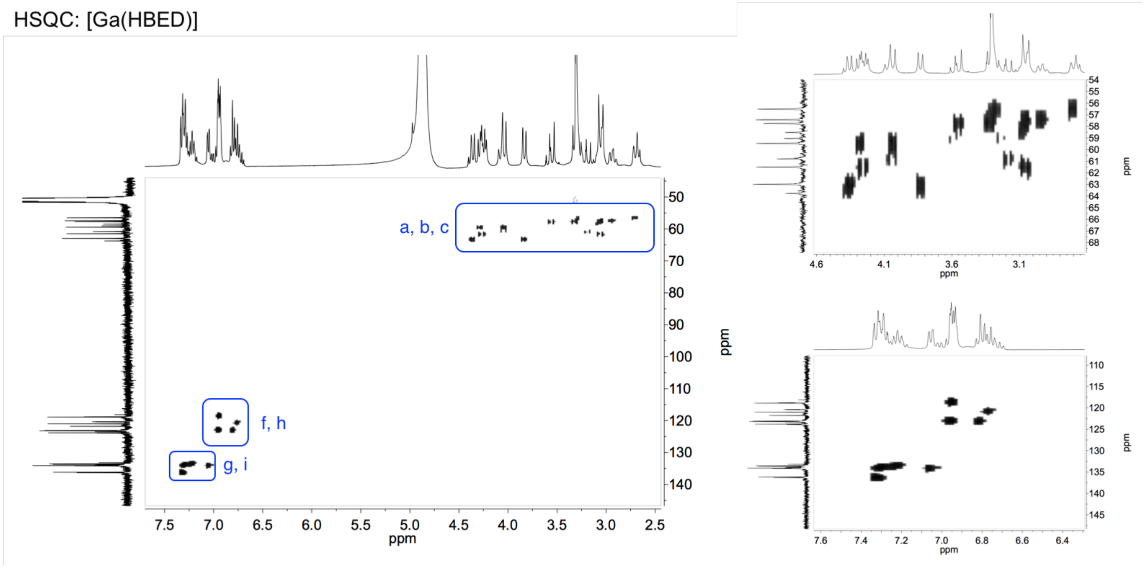

Figure S8. HSQC spectrum of [Ga(HBED)] in 50% D_2O / 50% CD_3OD (left) with expanded regions (right).

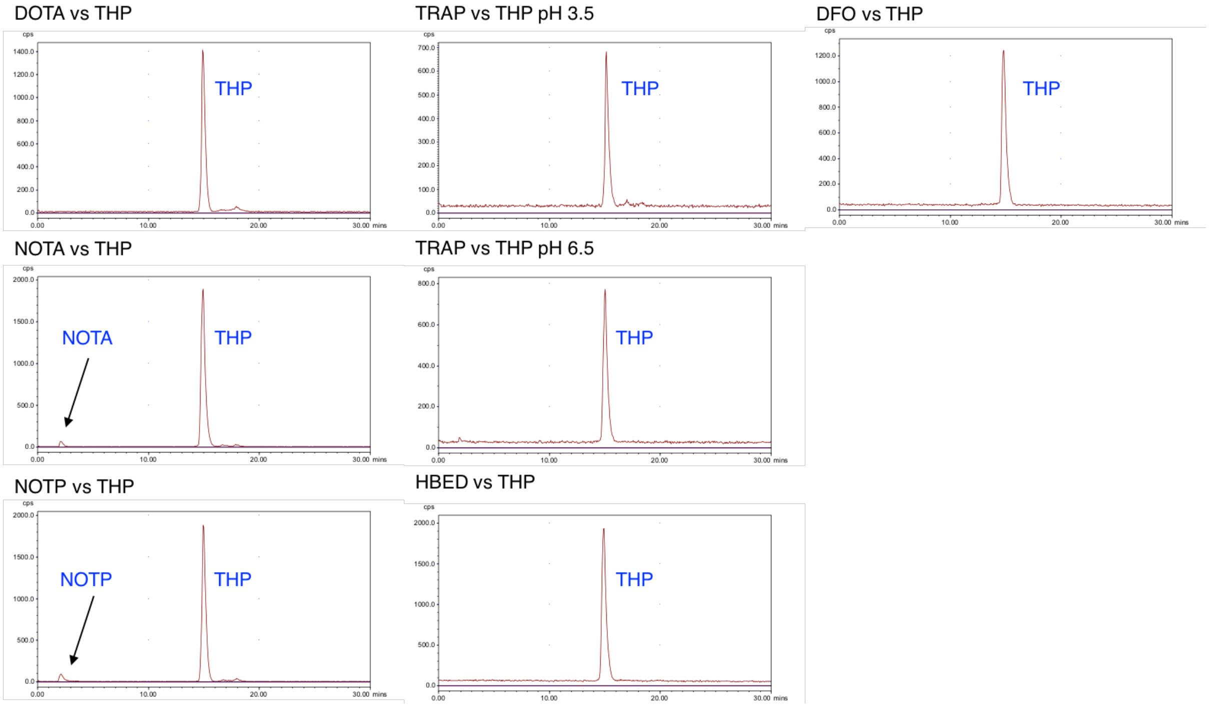

Figure S9. Exemplar reverse phase C_{18} HPLC chromatograms of solutions from $^{68}Ga^{3+}$ competition studies between THP and other chelators at 25 °C. ^{68}Ga-chelator radiochromatogram signals were assigned (blue labels) based on previously measured retention times (Figure S3). No significant differences were observed between reactions at pH 3.5 and pH 6.5 at 25 °C. Note: For reactions at 90 °C, the tacn derivatives NOTA, NOTP and TRAP competed more effectively with THP for $^{68}Ga^{3+}$ than at 25 °C (See Tables 2.1 and 2.2 of the manuscript, and Figure 5 of exemplar chromatograms in the manuscript), although the RCYs of [^{68}Ga(THP)] still exceeded 60% in high temperature reactions.

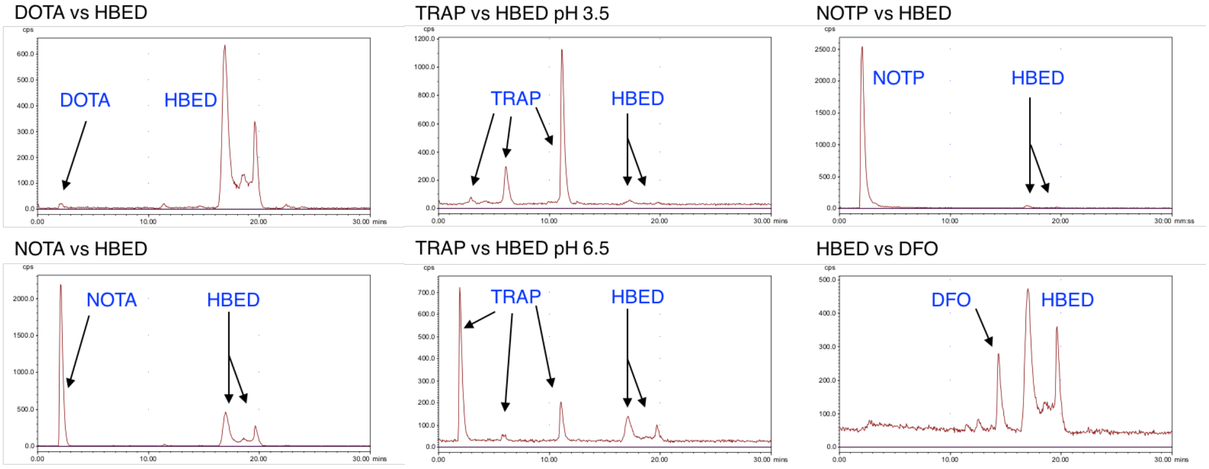

Figure S10. Exemplar reverse phase C_{18} HPLC chromatograms of solutions from $^{68}Ga^{3+}$ competition studies between HBED and other chelators at 25 °C. ^{68}Ga-chelator radiochromatogram signals were assigned (blue labels) based on previously measured retention times (Figure S3). No significant differences were observed between reactions at pH 3.5 and pH 6.5 at 25 °C, except in the case of reactions of HBED and TRAP (both sets of data are shown). At pH 6.5, HBED competed more effectively with TRAP for $^{68}Ga^{3+}$ than at pH 3.5.

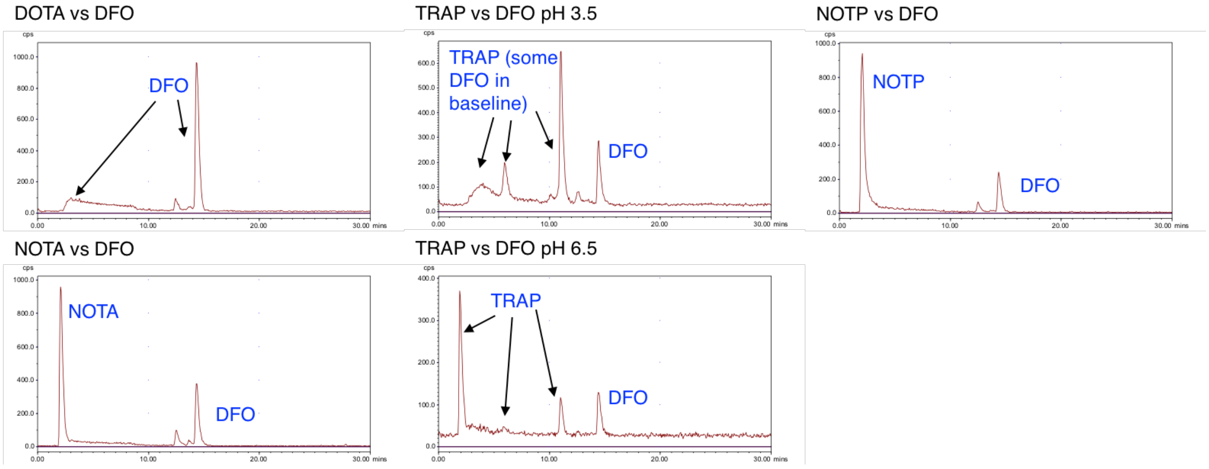

Figure S11. Exemplar reverse phase C_{18} HPLC chromatograms of solutions from $^{68}Ga^{3+}$ competition studies between DFO and other chelators at 25 °C. ^{68}Ga-chelator radiochromatogram signals were assigned (blue labels) based on previously measured retention times (Figure S3). No significant differences were observed between reactions at pH 3.5 and pH 6.5. Reactions were only undertaken at 25 °C, as some evidence of decomposition of DFO or [^{68}Ga(DFO)] was observed at higher temperatures.

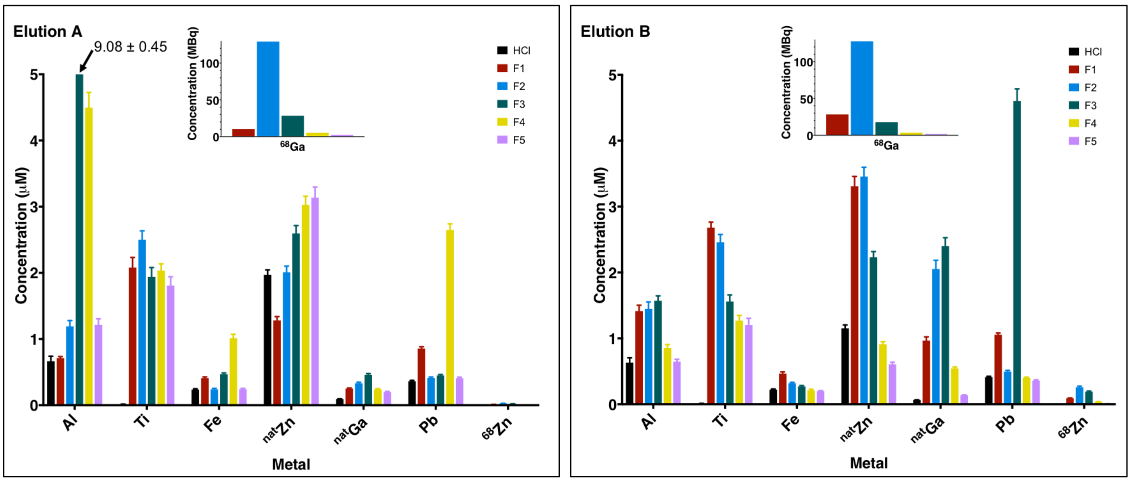

Figure 12. The concentrations of selected metals in ^{68}Ga generator eluate in all fractions (measured by ICP-MS). Elution **A** was obtained 5 hours after the previous elution, and elution **B**, 150 hours (five days) after the previous elution. Error bars represent standard deviation of the measurement (n = 5). ^{68}Zn concentrations correspond to ^{68}Zn arising from ^{68}Ga decay.

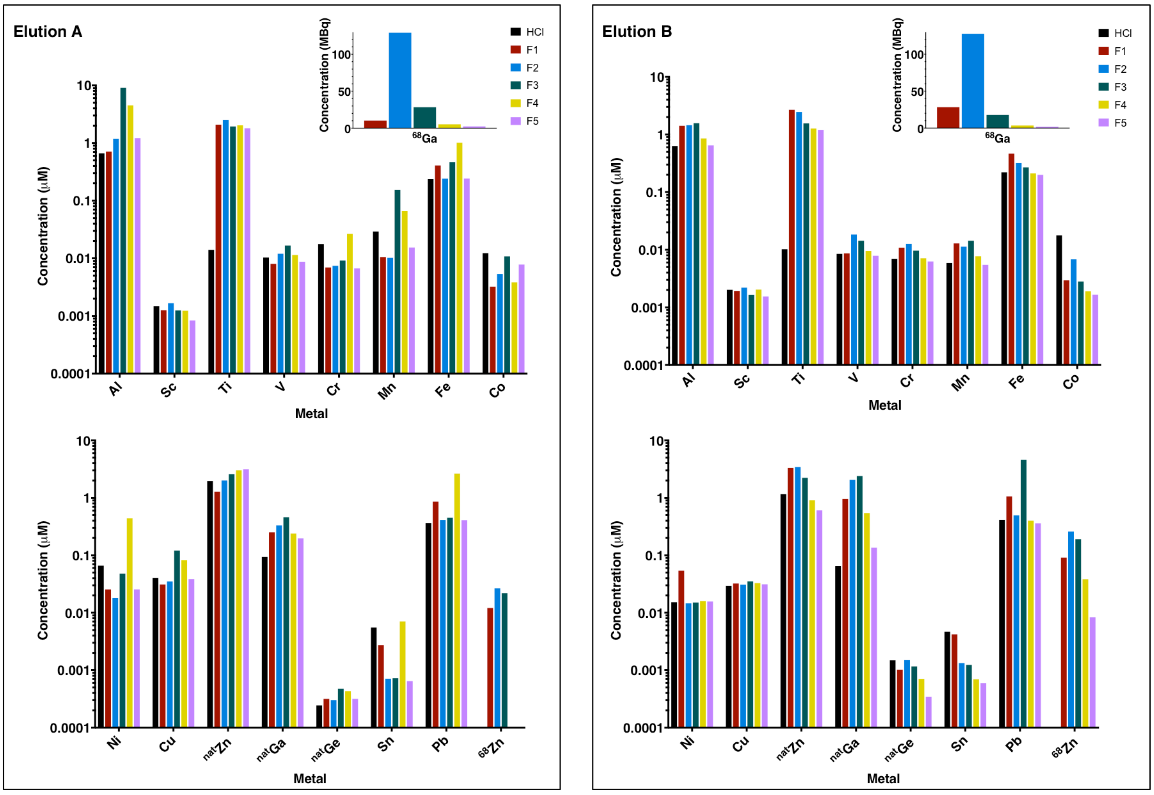

Figure S13. The concentrations of all analysed metals in ^{68}Ga generator eluate (measured by ICP-MS). Elution **A** was obtained 5 hours after the previous elution, and elution **B**, 150 hours (five days) after the previous elution.

Table S1. Crystallographic data

**[Ga(HBED)(H_2O)].CH_3CN**	
Identification code	xstr0762
formula	$C_{22}H_{26}GaN_3O_7$
formula weight	514.18
crystal system	monoclinic
space group	$P2_1/c$
a, b, c (Å)	12.96001 (14), 1.01939 (10), 25.0910 (3)
α, β, γ (°)	90, 97.7700 (10), 90
cell volume (Å^3)	2261.60 (5)
Z	4
temperature (K)	150.4 (5)
radiation	CuKα (λ = 1.54184)
reflections collected	34357
independent reflections collected	4541
R-factor (%)	2.92

Table S2. Selected bond lengths and angles of [Ga(HBED)(H_2O)].CH_3CN

Bond length (Å)	
Ga1 – O1	1.9757 (12)
Ga1 – O2	1.9437 (12)
Ga1 – O3	1.9743 (12)
Ga1 – O4	1.8615 (12)
Ga1 – N1	2.1009 (14)
Ga1 – N2	2.1973 (13)
**Bond angle (°)**	
N1 – Ga1 – N2	84.31 (5)
O1 – Ga1 – N2	88.52 (5)
O2 – Ga1 – O1	92.51 (5)
O2 – Ga1 – N1	95.53 (5)
O2 – Ga1 – N2	81.73 (5)
O3 – Ga1 – O1	88.01 (5)
O3 – Ga1 – N1	82.30 (5)
O3 – Ga1 – N2	89.09 (5)
O4 – Ga1 – O3	97.68 (5)
O4 – Ga1 – O1	92.48 (5)
O4 – Ga1 – O2	91.49 (5)
O4 – Ga1 – N1	95.78 (5)

Table S3. Mobile phase conditions for iTLC analyses

	**Mobile phase**
**(1)**	Aqueous sodium citrate (0.1 M, pH 5.5)
**(2)**	Aqueous sodium phosphate solution (0.4 M, pH 4)
**(3)**	Ammonium acetate solution (1 M in 80% methanol, 20% water)

Table S4. R_f values of [^{68}Ga(THP)], [^{68}Ga(DFO)] and unreacted ^{68}Ga in mobile phase 1

**Species**	**R_f value**
Soluble ^{68}Ga	> 0.9
^{68}Ga colloids	< 0.1
THP-Ac	< 0.1
DFO	< 0.1

Note: Based on quantification of ^{68}Ga colloid and soluble $^{68}Ga^{3+}$ in chelator-free reactions, RCYs for both [^{68}Ga(THP)] and [^{68}Ga(DFO)] were adjusted to account for coincident R_f values of ^{68}Ga colloid, [^{68}Ga(THP)] and [^{68}Ga(DFO)]. In chelator-free reactions, at pH 3.5, 25 °C, ^{68}Ga colloid = 12±2%; pH 6.5, 25 °C, ^{68}Ga colloid = 13±4%; pH 3.5, 90 °C, ^{68}Ga colloid = 16±7%; pH 6.5, 90 °C, ^{68}Ga colloid = 5±1%.

Table S5. R_f values of [^{68}Ga(DOTA)], [^{68}Ga(NOTA)], [^{68}Ga(NOTP)], [^{68}Ga(TRAP)] and [^{68}Ga(HBED)] and unreacted ^{68}Ga in mobile phase 2

Species	R_f value
Unreacted ^{68}Ga at pH 3.5, 90 °C	0 – 0.1 and 0.7 – 0.9
Unreacted ^{68}Ga under other conditions	< 0.1
[^{68}Ga(DOTA)]	0.8 – 1
[^{68}Ga(NOTA)]	0.8 – 1
[^{68}Ga(NOTP)]	0.6 – 0.7
[^{68}Ga(TRAP)]	0.8 – 1
[^{68}Ga(HBED)]	0.8 – 1

Note (a): Temperature-dependant differences in R_f of non-chelated $^{68}Ga^{3+}$ using mobile phase 2 indicate that the speciation of Ga^{3+} at pH 3.5 is dependent on the temperature at which the radiolabelling is carried out. Existing assumptions about Ga speciation based on data acquired under different conditions to those used in radiolabelling (temperature, buffers, presence of other adventitious metal ions with their own complex speciation) do not adequately describe radiolabelling systems. It was beyond the scope of this study to probe the speciation of Ga^{3+} at different temperatures, so this was not pursued further.

Note (b): For reactions undertake at pH 3.5 and 90 °C, non-chelated $^{68}Ga^{3+}$ could not be distinguished from [^{68}Ga(chelator)] using mobile phase (2) (except in the case of [^{68}Ga(NOTP)]). Thus, a third mobile phase that allowed distinction of unreacted ^{68}Ga from [^{68}Ga(chelator)] was employed (mobile phase 3).

Table S6. R_f values of [^{68}Ga(DOTA)], [^{68}Ga(NOTA)], [^{68}Ga(NOTP)], [^{68}Ga(TRAP)] and [^{68}Ga(HBED)] and unreacted ^{68}Ga in mobile phase 3 (for reactions undertaken at pH 3.5, 90 °C)

Species	R_f value
Unreacted ^{68}Ga	< 0.3
[^{68}Ga(DOTA)]	0.65 – 0.75
[^{68}Ga(NOTA)]	0.8 – 0.9
[^{68}Ga(NOTP)]	0.3 – 0.4
[^{68}Ga(TRAP)]	0.4 – 0.6
[^{68}Ga(HBED)]	0.9 – 1
